# Supplementary material for: Acceptability of bisphosphonates among patients, clinicians and managers: a systematic review and framework synthesis
Source: BMJ Open. 2020 Nov 3;10(11):e040634. doi: 10.1136/bmjopen-2020-040634 (PMC7640526; doi:10.1136/bmjopen-2020-040634)
Supplement: Supplementary data [file bmjopen-2020-040634supp002.pdf]

Supplementary Material 1. OVID MEDLINE Search strategy

For Ovid: The following table is an explanation of the symbols used in the search strategy below.

/	indicates an index term (MeSH/EMTREE heading).
exp	before an index term indicates that all subheadings were selected.
af.	Indicates a search for a term in all fields.
.ti,ab,kf.	indicates a search for a term in title/abstract/word(s) in keyword [MEDLINE].
mp.	indicates a search for a term in 'multi-purpose' fields, including the title, abstract, floating sub-heading word, keyword heading word, subject heading word.
tw.	Indicates a search for a term in title and abstract.
$	at the end of a term indicates that this term has been truncated.
?	optional wild card character replaces zero or one character within a word or at the end of a word
adj	indicates a search for tw.o terms where they appear adjacent to each another
adj*n*	indicates a search for two terms where they appear within *n* words of each another

	Searches
1	diphosphonates/ or alendronate/ or ibandronic acid/ or risedronic acid/ or zoledronic acid/ or etidronic acid/ or pamidronate/
2	diphosphon$.ti,ab,kf.
3	bisphosphon$.ti,ab,kf.
4	alendron$.ti,ab,kf.
5	fosamax.ti,ab,kf.
6	risedron$.ti,ab,kf.
7	actonel.ti,ab,kf.
8	zoledron$.ti,ab,kf.
9	aclasta.ti,ab,kf.
10	ibandron$.ti,ab,kf.
11	etidron$.ti,ab,kf.
12	pamidron$.ti,ab,kf.
13	or/1-12
14	attitude/
15	attitude of health personnel/
16	exp attitude to health/ [includes patient satisfaction and patient preference]
17	choice behavior/
18	decision making/
19	attitud$.ti,ab,kf.
20	percept$.ti,ab,kf.
21	expectation$.ti,ab,kf.

22	experienc$.ti,ab,kf.
23	preferen$.ti,ab,kf.
24	choice$.ti,ab,kf.
25	belie$.ti,ab,kf.
26	opinion$.ti,ab,kf.
27	priorit$.ti,ab,kf.
28	benefi$.ti,ab,kf.
29	reason$.ti,ab,kf.
30	decision$.ti,ab,kf.
31	motiv$.ti,ab,kf.
32	justif$.ti,ab,kf.
33	(concern or concerns or concerned).ti,ab,kf.
34	(view or views or viewed).ti,ab,kf.
35	satisf$.ti,ab,kf.
36	value$1.ti,ab,kf.
37	or/14-36
38	Qualitative Research/ [After DeJean et al., 2016. Qual Health Res **26**(10): 1307-1317]
39	interview/
40	(theme$ or thematic).mp.
41	qualitative.af.
42	nursing methodology research/
43	questionnaire$.mp.
44	ethnological research.mp.
45	ethnograph$.mp.
46	ethnonursing.af.
47	phenomenol$.af.
48	(grounded adj (theor$ or study or studies or research or analys?s)).af.
49	(life stor$ or women$ stor$).mp.
50	(emic or etic or hermeneutic$ or heuristic$ or semiotic$).af.
51	((data adj1 saturat$) or participant observ$).tw.
52	(social construct$ or postmodern$ or post modern$ or poststructural$ or post structural$ or feminis$ or interpret$).mp.
53	(action research or cooperative inquir$ or co operative inquir$).mp.
54	(humanistic or existential or experiential or paradigm$).mp.

55	(field adj (study or studies or research)).tw.
56	human science.tw.
57	biographical method.tw.
58	theoretical sampl$.af.
59	((purpos$ adj4 sampl$) or (focus adj group$)).af.
60	(account or accounts or unstructured or open ended or text$ or narrative$).mp.
61	(life world or conversation analys?s or personal experience$ or theoretical saturation).mp.
62	((lived or life) adj experience$).mp.
63	cluster sampl$.mp.
64	observational method$.af.
65	content analysis.af.
66	(constant adj (comparative or comparison)).af.
67	((discourse$ or discurs$) adj3 analys?s).tw.
68	narrative analys?s.af.
69	heidegger$.tw.
70	colaizzi$.tw.
71	spiegelberg$.tw.
72	van manen$.tw.
73	van kaam$.tw.
74	merleau ponty.tw.
75	husserl$.tw.
76	foucault$.tw.
77	(corbin$ adj2 strauss$).tw.
78	glaser$.tw.
79	(mix$ adj2 (method$ or design$)).af. [filter amended to identify mixed method studies]
80	or/38-79
81	13 and 37 and 80

Supplementary Material 2. CASP Quality Appraisal Checklist

All ten questions answered with one of four options: Yes, unsure, partial, or No

Section A: Are the results of the study valid?

1. Was there a clear statement of the aims of the research?
2. Is a qualitative methodology appropriate?
3. Was the research design appropriate to address the aims of the research?
4. Was the recruitment strategy appropriate to the aims of the research?
5. Was the data collected in a way that addressed the research issue?
6. Has the relationship between researcher and participants been adequately considered?

Section B: What are the results?

7. Have ethical issues been taken into consideration?
8. Was the data analysis sufficiently rigorous?
9. Is there a clear statement of findings?

Section C: Will the results help locally?

10. How valuable is the research?

Supplementary Material 3. Subtheme descriptions and illustrative key findings

Main theme	Subtheme	Description	Illustrative Key findings
Intervention Coherence	Necessity	Both patient and clinician participants described osteoporosis, falling and fracturing as a normal part of ageing and this view was associated with the perception that medication or treatment was futile.[16,38] One GP described the 'problem is not with the treatment, it's with the diagnosis': perceiving that the indications for treatment had broadened over recent years.[42] The absence of symptoms was reported by clinicians as a disincentive to patients accepting treatment,[33,36] however, patients questioned whether osteoporosis really was asymptomatic.[23] Patient participants who conceptualised osteoporosis as having consequences, e.g. as a cause of disability including 'shrinking' and 'stooping', were motivated to take medication.[37] Patient participants described other ways of controlling their condition and preventing fracture, for example, by not falling.[35]<br>In some patients who initiated treatment, the notion of osteoporosis as a chronic disease was noted not to make sense with the need to take bisphosphonate medication for 5 years.[23]	Patients perceived minimal susceptibility to the negative consequence of osteoporosis in the future and did not consider osteoporosis to be a serious health condition.[30]<br>Avoiding consequences (including shrinking, stooping, fractures) of osteoporosis was a strong motivator for adherence in PMW.[37]
	Concerns	Before starting bisphosphonates, patients noted concern and fear of bisphosphonate-specific side effects. This could be informed by vicarious experience of a family member,[43] or information from the media.[37] The special instructions for use, the limited duration of treatment and the name 'acid' were all cited as reasons underlying the perception that bisphosphonates must be harmful. Both patients and HCP's also cited a mistrust of pharmaceutical companies,[33,38,42] or a general aversion to drugs.[35,37,39,42]	{Women} were concerned about the long lists of drug side effects in advertisements.[16]<br>'Once you're on it, then it stays in your system and you wonder what damage have you've done to yourself?'[16]<br>Some PMW did not like the idea of taking any medications because they viewed medications as artificial and thought they had unpredictable effects.[37]
	Perceptions of own health	Some patients reported a perception that they were healthy, with some disbelieving they had osteoporosis and/or high fracture risk, and therefore and would reject medication and a label of a disease.[37] Conversely, others conceptualised bisphosphonates as a	Some patients initiated bisphosphonates to stay healthy.[41]<br>For PMW who considered themselves healthy, the idea of

		mechanism to remain healthy[41] and/or autonomous.[38] In a study of French GPs, on respondent also suggested patients wanted to know how to '"age well".[33]	medication was disconcerting as it meant perceiving themselves as sick.[37]
	Decision process	Across studies patients and HCPs described perceptions that the benefits did not outweigh the risks.[16,29,35,41,] Often in these descriptions, the value of treatment was not clearly articulated meaning this assessment meant the patient weighing up staying as they were, or experiencing new side effects.[38] However, even when the risk of fracture was acknowledged, medication could still be seen as something to avoid.[35] The opposing view that the 'benefits were worth the costs' was evident in circumstances where benefits were described.[37] Others studies with patients reported that this decision was 'difficult' with one participant describing it as like 'Russian roulette'.[44] Balancing necessity against concerns was influenced by contingent factors such as trust in the clinician and could either be an easy or difficult and ongoing process. Patient participants talked about 'confidence' and 'trust' in their HCP, which could be associated with minimal contemplation to take treatment, or alternatively mistrust, or a failure to be 'convinced'.[16,25,26,37,40] Some patients reported clinicians as being persistent in their recommendation to take bisphosphonates;[40] however, conversely, patients also described by dissuaded by their doctor against treatment.[32] Often, patients described seeking information from other sources to make the final decision which often resulted in a decision against treatment.[44]<br>For those who initiated medication, an ongoing re-assessment of risk and benefit was noted,[23,41,44] particularly in studies that employed longitudinal methods.[27,38, 41] Patients reported their decision making was influenced by experiencing a future fracture,[44] follow-up scans,[25] experienced side effects,[37,38] views of others and other experienced illnesses or life events.[27]	For some, the decision to take bisphosphonate involved minimal contemplation because they liked/trusted their health care provider.[44]<br>Patients who found the decision difficult sought alternative sources of information (professional and non) which often resulted in decision not to take OP medication.[44]
	Ethicality	Both orthopaedic and primary care clinicians reported a 'bias' against treating the elderly due to a belief 'nothing can be done for them'.[16] However, some patients also perceived that they were too old to benefit.[35] HCPs were seen to use the using ethical principle of non-maleficence to justify not recommending bisphosphonates. They questioned the negative side effects 'for a benefit that has not really been proven' and worried about being blamed for causing their patients ill-health.[28,40] Patients, in some circumstances, doubted the beneficence of the health care professionals e.g. perceiving	Clinicians {primary care and specialists} report bias against treating elderly patients because of a general tendency to believe that nothing can be done for them.[16]

		their physician as a 'pill pusher' or the motivation for prescribing medication being to receive money in return.[40]	
Affective Attitudes	Emotions	Patients described wide-ranging fears including fear of common and rare side effects and fear of new side effects emerging in the future. Patients described fear of bisphosphonates staying in their system,[16] with one patient participant describing bisphosphonates as akin to chemicals used to clean machines.[23] Patients also worried information was being withheld, or were fearful of the sheer amount of information to take in.[37] Both clinicians and patients described media reports as the source of fear, with patients also citing experiences of friends and family.[37] Fear of addiction was mentioned by patients in one study.[23] Patients and HCPs also expressed annoyance with the special instructions associated with oral bisphosphonate use, and annoyance with experienced oesophageal side effects.[40]<br>In two studies, patient participants reported that they experienced feelings of safety and reassurance when taking bisphosphonates,[26] linked to the anticipated benefits.[37]	"..when I read the side effects it was like a *horror film* really".[38]<br><br>medication provided a feeling of safety and reassurance.[26]
Burden	Special instructions	The method of administration of oral bisphosphonates caused concern to patients, both prior to initiating treatment,[42] and whilst on the treatment,[32] causing disruption to daily life. The need to remain upright after taking the medication and only being allowed to drink water was burdensome, and led to some disregarding the administration requirements.[37] Specific activities that needed to be actioned first thing in the morning also competed with taking oral bisphosphonates, with patients citing examples such as the need to have a coffee or run a family errand early every morning.[44] Primary care physicians reported that taking bisphosphonates was a 'hassle' for patients.[16] The frequency of the oral bisphosphonates, once a week, led to a number of reports of patients forgetting to take their medication.[16,23,37-39] Varying reports were identified about whether daily or weekly regimes were more or less burdensome.[16,37] Four studies reported patients' perceptions that the special instructions were not disruptive or burdensome.[26,27,37,39]	Some patients were able to rearrange their daily routines to accommodate {bisphosphonate} requirements, but others would intentionally disregard the administration requirements or forget to take the medication if it did not fit into their schedules.[37]
	Side effects	Experienced side effects were discussed in three of the studies interviewing clinicians,[28,29,31] eight with patients[23,26,27,37,38,41,44,45] and five with mixed participants.[16,35,39,42,43] Experienced side effects were reported as a common reason for lack of adherence, with gastrointestinal disturbances being described as	Gastrointestinal disturbances from taking bisphosphonates were most notable and were described as

		"horrendous diarrhoea" and "wrecking my stomach.[37,39] Patients reported stopping medications after experiencing side effects, did not always disclose side effects to HCPs and noted that the treatment 'was almost more disabling than the disease'.[27,32,46]	"horrendous diarrhoea" and "wrecking my stomach.[37]
	Costs	Financial costs were discussed in five studies, four of which were conducted in North America and one in Australia.[16,28,37,43,46] Patients did not report cost as a barrier to bisphosphonates specifically, however, medical insurance was perceived by clinicians as a barrier due to its complexity.[29,39,43] Indirect costs relating to travel and the need for increased dental checks were mentioned briefly but not described as a problem.[45,46]	Cost was not a limiting factor to adherence if patients had insurance coverage for medications. Even patients without insurance expressed a willingness to make sacrifices to pay for the medications because they thought the benefits were worth the cost.[37]<br>Providers {secondary care} stated that due to cost not being covered by insurance companies, patients stop taking or alter dose/frequency.[39]
Opportunity costs	Co-morbid conditions	Physicians perceived bisphosphonate treatment was less important to patients who might have other more pressing health conditions [29,45] particularly in the absence of symptoms.[27,33] Patients also reported that other health conditions took priority over their prescribed bisphosphate leading them not to start or discontinue medication.[32] Within the time-limited consultation, multiple competing priorities relating to other health conditions was reported by HCPs, resulting in a 'pecking order', and less time to discuss bisphosphonates.[35,45]	(Bisphosphonates) are lower down in the pecking order of things that we look at when we are supervising polypharmacy, when we are looking at chronic disease management".[45]
Perceived effectiveness	Mechanism of effectiveness	*Mechanism of effectiveness:* Patients expressed confusion about how bisphosphonates work and uncertainty about whether they strengthen, prevent worsening or slow the decline in bone density.[25,26,39] Patients talked about bone density scans as providing 'proof' of whether their medication was effective, however, there were differing reports of whether stabilisation in density was considered as treatment success.[35,40] The lack of systematic reduction in fracture or improvement in bone density was noted to result in ambivalence about efficacy and importance.[35] Patients described wanting more explanation about, and evidence of effectiveness (including quantified benefit).[16,23,37,38,40] Prior to initiating treatment, the perceived effectiveness of bisphosphonates was influenced in patients primarily by vicarious experience of friends	Taking anti-osteoporosis drugs was noted to not always seem to lead to improvement in their bone density and did not systematically prevent fracture.[35]

		or relatives.[40,42,43] Examples of relatives who had fractured on treatment or had hip or knee joint replacements were given as examples of lack of efficacy.[42]<br>Patients cited clinicians not meeting their informational needs about effectiveness, which may have been due to their own reported doubts.[29,42] Other clinicians expressed continued doubts about effectiveness in specific populations (e.g. the elderly) or in in relation to fracture risk at specific sites.[35] Patients in one study reported being told by health care professionals bisphosphonates are not effective for everyone[24] and in one study, clinicians questioned predictors of response.[29]	
	Monitoring and follow-up	Follow-up and monitoring were reported by clinicians[34] and patients[38] to support adherence to oral treatment, but generally felt to be lacking in primary care, in part due to uncertainties about who, when and what to monitor.[34] Patients reported not feeling supported with continued persistence with treatment[38] and reported the need for more reviews, feedback and help with 'ways to keep going' with medications.[16,23,38]	Women anticipated the next DXA scan as being the "proof" of whether the treatment was effective.[2731] Reviewing patients' BMD results with them helped them evaluate the status of their osteoporosis, which motivated them to either start or continue taking their medicine.[37]
Self-efficacy	Supporting routinisation	*Supporting routinisation* Being able to successfully follow the special instructions for taking oral bisphosphonates, and incorporate the regime into daily routines appeared to be important to acceptability.[39] Other reported strategies to support self-efficacy were using pill compartments and calendar systems/reminders.[16] Patients reported that HCPs should supplement their oral instructions about BP administration with written ones.[39] Information, support and encouragement was needed throughout treatment but felt to be lacking by patients[16,38,44]. Patients and HCPs reported insufficient time in consultations to cover all the information about bisphosphonate medication.[35,39]	Patients noted that tips for routinizing medication use, such as using triggers (e.g., meals, calendars, placement of medications) to remember when to take medications, facilitated long-term adherence.[16]
	HCP knowledge and attitudes	Primary care providers did not feel confident in their own knowledge about bisphosphonates; they described guidelines as confusing and too detailed, expressing a number of uncertainties relating to who to start medication in, how long to continue medication for, the relationship between bisphosphonates and co-dependency for calcium/vitamin D, safety, when treatment should be changed including dose.[16,25,29,35] Some primary care clinicians indirectly suggested perceptions that osteoporosis was not a priority. Secondary care providers suggested osteoporosis champions in primary care would help educate primary care clinicians who were less interested in the condition.[39,45] It was also reported that non-medical clinicians	Physicians reported need for training in treating and help with therapeutic decision making.[35]

		(pharmacists or nurses) may be more knowledgeable or have more time to discuss bisphosphonates.[39,45]	
	Service level barriers	In terms of professional roles, clinicians in two studies described uncertainty about whose role it was to start and monitor treatment.[16,34] This was compounded by perceived poor communication between primary and secondary care, including update of the patients prescriptions on the electronic medical record.[39] Further reported barriers to treatment included lack of incentivisation[34] difficulty ordering, accessing or interpreting investigations to monitor treatment,[16,29] external restrictions on prescribing and access to intravenous bisphosphonates[34] and lack of time in primary care consultations.[16]	Provider barriers to treatment include lack of knowledge, other priorities, limited access and limited time.[36]<br>GPs regretted the absence of consensus about the professional in charge of osteoporosis.[32]<br>A number of participants {HCPs/managers} thought that intravenous zoledronic acid should be more widely available to improve adherence.[34]
