# Supplementary material for: Acceptability of bisphosphonates among patients, clinicians and managers: a systematic review and framework synthesis
Source: BMJ Open. 2020 Nov 3;10(11):e040634. doi: 10.1136/bmjopen-2020-040634 (PMC7640526; doi:10.1136/bmjopen-2020-040634)
Supplement: Author's manuscript [file bmjopen-2020-040634.draft_revisions.pdf]

BMJ Open is committed to open peer review. As part of this commitment we make the peer review history of every article we publish publicly available.

When an article is published we post the peer reviewers' comments and the authors' responses online. We also post the versions of the paper that were used during peer review. These are the versions that the peer review comments apply to.

The versions of the paper that follow are the versions that were submitted during the peer review process. They are not the versions of record or the final published versions. They should not be cited or distributed as the published version of this manuscript.

BMJ Open is an open access journal and the full, final, typeset and author-corrected version of record of the manuscript is available on our site with no access controls, subscription charges or pay-per-view fees (http://bmjopen.bmj.com).

If you have any questions on BMJ Open's open peer review process please email info.bmjopen@bmj.com

BMJ Open

Acceptability of bisphosphonates among patients, clinicians and managers: a systematic review and framework synthesis.

Journal:	*BMJ Open*
Manuscript ID	bmjopen-2020-040634
Article Type:	Original research
Date Submitted by the Author:	18-May-2020

Complete List of Authors:	Paskins, Zoe; Keele University, School of Primary Community and Social Care; Haywood Hospital, Academic Rheumatology Centre<br>Crawford-Manning, Fay; Keele University, School of Primary Community and Social Care; Haywood Hospital, Academic Rheumatology Centre<br>Cottrell, Elizabeth ; Keele University, School of Primary, Community and Social Care<br>Corp, Nadia; Keele University, School of Primary, Community and Social Care<br>Wright, Jenny; Keele University, School of Primary Community and Social Care<br>Jinks, Clare; Keele University, School of Primary, Community and Social Care<br>Bishop, Simon; University of Nottingham, Centre for Health Innovation, Leadership and Learning<br>Doyle, Alison; Royal Osteoporosis Society<br>Ong, Terence; University of Malaya, Faculty of Medicine<br>GITTOES, NEIL; University of Birmingham, Centre for Endocrinology Diabetes and Metabolism<br>Leonardi-Bee, Jo; University of Nottingham, Faculty of Medicine & Health Sciences<br>Langley, Tessa; University of Nottingham, Division of Epidemiology and Public Health<br>Horne, Robert; University College London, School of Pharmacy<br>Sahota, Opinder; Nottingham University Hospitals NHS Trust, Department of Geriatric Medicine

Keywords:	Rheumatology < INTERNAL MEDICINE, Bone diseases < ORTHOPAEDIC & TRAUMA SURGERY, Musculoskeletal disorders < ORTHOPAEDIC & TRAUMA SURGERY

SCHOLARONE™
Manuscripts

I, the Submitting Author has the right to grant and does grant on behalf of all authors of the Work (as defined in the below author licence), an exclusive licence and/or a non-exclusive licence for contributions from authors who are: i) UK Crown employees; ii) where BMJ has agreed a CC-BY licence shall apply, and/or iii) in accordance with the terms applicable for US Federal Government officers or employees acting as part of their official duties; on a worldwide, perpetual, irrevocable, royalty-free basis to BMJ Publishing Group Ltd ("BMJ") its licensees and where the relevant Journal is co-owned by BMJ to the co-owners of the Journal, to publish the Work in this journal and any other BMJ products and to exploit all rights, as set out in our licence.

The Submitting Author accepts and understands that any supply made under these terms is made by BMJ to the Submitting Author unless you are acting as an employee on behalf of your employer or a postgraduate student of an affiliated institution which is paying any applicable article publishing charge ("APC") for Open Access articles. Where the Submitting Author wishes to make the Work available on an Open Access basis (and intends to pay the relevant APC), the terms of reuse of such Open Access shall be governed by a Creative Commons licence – details of these licences and which Creative Commons licence will apply to this Work are set out in our licence referred to above.

Other than as permitted in any relevant BMJ Author's Self Archiving Policies, I confirm this Work has not been accepted for publication elsewhere, is not being considered for publication elsewhere and does not duplicate material already published. I confirm all authors consent to publication of this Work and authorise the granting of this licence.

For peer review only

Title: **Acceptability of bisphosphonates among patients, clinicians and managers: a systematic review and framework synthesis.**

Corresponding Author: Dr. Zoe Paskins, School of Primary Community and Social Care, Keele University, David Weatherall Building, Newcastle-under-Lyme, UK, ST5 5BG and Haywood Academic Rheumatology Centre, Haywood Hospital, High Lane, Burslem, Stoke-on-Trent ST6 7AG.

z.paskins@keele.ac.uk, 01782 733975

Author List:

Dr. Zoe Paskins, School of Primary, Community and Social Care, Keele University & Haywood Academic Rheumatology Centre, Stoke-on-Trent, UK

Dr. Fay Crawford-Manning, School of Primary, Community and Social Care, Keele University & Haywood Academic Rheumatology Centre, Stoke-on-Trent, UK

Dr. Elizabeth Cottrell, School of Primary, Community and Social Care, Keele University, Newcastle-under-Lyme, UK

Dr. Nadia Corp, School of Primary, Community and Social Care, Keele University, Newcastle-under-Lyme, UK

Dr. Jenny Wright, School of Primary, Community and Social Care, Keele University, Newcastle-under-Lyme, UK

Prof. Clare Jinks, School of Primary, Community and Social Care, Keele University, Newcastle-under-Lyme, UK

Dr. Simon Bishop, Centre for Health Innovation, Leadership and Learning, University of Nottingham, Nottingham, UK

Alison Doyle, Royal Osteoporosis Society, Bath, UK

Dr. Terence Ong, Department of Medicine, Faculty of Medicine, University of Malaya, Kuala Lumpar, Malaysia

For peer review only

Prof. Neil Gittoes, Centre for Endocrinology Diabetes and Metabolism, University of Birmingham, Birmingham, UK

Prof. Jo Leonardi-Bee, Faculty of Medicine & Health Sciences, University of Nottingham, Nottingham, UK

Associate Prof. Tessa Langley, Division of Epidemiology and Public Health, University of Nottingham, Nottingham, UK

Prof. Robert Horne, School of Pharmacy, University College London, London, UK

Prof. Opinder Sahota, Department of Geriatric Medicine, Nottingham University Hospitals NHS Trust, Nottingham, UK

Author Statement:

Conceptualisation: ZP, SB, EC, AD, TO, NG, JLB, TL, OS. Protocol: ZP, EC, NC, SB, LLB, TL, TO, OS, NG, AD. Search Implementation: ZP, EC, NC, JW, FCM. Data extraction and Quality: ZP, EC, NC, JW, FCM, SB. Synthesis: ZB, SB, EC, FCM, RH, CJ. Writing- Original draft: ZP, FCM. Writing- Review and Editing: ZP, SB, AD, TO, NG, JLB, CJ, TL, OS, FCM, JW, NC, NH, EC

Key Words:

Acceptability; Theoretical Framework of Acceptability, Bisphosphonates; Osteoporosis

Current word count 3875

For peer review only

Abstract (299/300 words):

OBJECTIVE: To explore the acceptability of different bisphosphonate regimens for the treatment of osteoporosis among patients, clinicians and managers, payers and academics.

DESIGN: A systematic review of primary qualitative studies. Seven databases were searched from inception to July 2019. Screening, data extraction and quality assessment of full-articles selected for inclusion were performed independently by two authors. A framework synthesis was applied to extracted data based on the Theoretical Framework of Acceptability (TFA). The TFA includes seven domains relating to sense-making, emotions, opportunity costs, burden, perceived effectiveness, ethicality and self-efficacy. Confidence in synthesis findings was assessed.

SETTING: Any developed country healthcare setting

PARTICIPANTS: Patients, healthcare professionals, managers, payers and academics

INTERVENTION: Experiences and views of oral and intravenous bisphosphonates.

RESULTS: Twenty-five studies were included, mostly describing perceptions of oral bisphosphonates. We identified, with high confidence, how patients and HCPs make sense (coherence) of bisphosphonates by balancing perceptions of need against concerns, how uncertainty prevails about bisphosphonate perceived effectiveness and a number of individual and service factors that have potential to increase self-efficacy in recommending and adhering to bisphosphonates. We identified, with moderate confidence, that bisphosphonate taking induces concern, but has the potential to engender reassurance, and that both side effects and special instructions for taking oral bisphosphonates can result in treatment burden. Finally, we identified with low confidence that multi-morbidity plays a role in people's perception of bisphosphonate acceptability.

CONCLUSION: By using the lens of acceptability, our findings demonstrate with high confidence that a theoretically informed, whole-system approach is necessary to both understand and improve adherence. Clinicians and patients need supporting to understand the need for bisphosphonates, and clinicians need to clarify to patients what constitutes bisphosphonate treatment success. Further research is

needed to explore perspectives of male patients and those with multi-morbidity receiving bisphosphonates, and patients receiving intravenous treatment.

Strengths and Limitations

- Comprehensive search underpinned by theoretical framework

- Inclusion of clinician views in addition to patient perspectives.

- Use of GRADE-CERQual to give confidence in findings

- Lack of clarity on bisphosphonate regimens patients were taking in some included studies.

INTRODUCTION/BACKGROUND

Osteoporosis is a disease that is characterised by skeletal fragility and changes in bone microarchitecture resulting in increased risk of fractures with no or low trauma.[1] The management and care of people with low trauma or fragility fractures results in considerable societal economic burden, annual cost in the UK alone is £4.4 billion.[2] Furthermore, the personal impact of fragility fractures is considerable, with potential deleterious effects on physical and psychological health, ability to live independently and increased risk of death. Many of these fractures are potentially preventable with appropriate cost- and clinically-effective drug treatments such as bisphosphonates, the mainstay of osteoporosis treatment. However, the success of treatment depends on patients initiating (starting), executing (or implementing - taking correctly) and persisting (continuing) medication; collectively these processes are described as adherence. Adherence with osteoporosis medications is notoriously poor and reported to be poorer than other disease areas. Oral bisphosphonate persistence rates at 1 year are commonly estimated between 16 and 60%.[3] Worldwide, many people who would benefit from osteoporosis drugs are not receiving them, and this treatment gap has been described as an ‘osteoporosis crisis’.[4] The treatment gap is compounded by poor adherence which results in potentially preventable fragility fractures with their associated burden for patients and their carers, difficulties in professional-patient relationships, and wasted healthcare resources.[5]

For peer review only

There are a number of different bisphosphonates, some are administered orally others intravenously. A variety of regimes in terms of dose frequency also exists. Alendronic acid, an oral once-weekly bisphosphonate, is considered first-line and most commonly used.[6] Bisphosphonates work to reduce fracture risk. A recent network meta-analysis demonstrated that bisphosphonate treatment reduces the risk of fragility fracture (depending on site) by 33-54%.[6] Oesophageal or gastrointestinal related side effects are the most common adverse effects of oral bisphosphonate use. To counter these, patients taking oral bisphosphonates are required to remain upright and fast for half an hour after ingestion. Rare side effects of bisphosphonates include osteonecrosis of the jaw and atypical femur fractures, both of which have received significant media attention. Such media reports are temporally related to declining bisphosphonate use.[7] Due to the gastrointestinal side effects and special instructions for taking oral treatment, it has been suggested that alternative bisphosphonate regimens, for example annual intravenous zoledronic acid, may promote long-term adherence.[8-11] Studies to date which have examined patient preferences for osteoporosis treatment, suggest that patients prefer injections given less frequently;[12-14] however, research in other chronic diseases shows that although adherence is improved with less frequent medications and that patients prefer oral to injection treatment.[15] In osteoporosis, the majority of studies that explore patient preferences employ quantitative methods, for example, discrete choice experiments where patients are asked to choose between hypothetical treatments in regards to various attributes (e.g. efficacy, side effects, route and frequency of administration).[13] Such studies cannot provide comprehensive insight into patient views, experiences or the explanations for these preferences.

In order to fully understand the osteoporosis treatment gap, and ultimately improve adherence, it is important to understand perspectives of all relevant stakeholders: patients, healthcare professionals (HCPs), managers, payors and academics.[16,17] This can be achieved using the lens of ‘acceptability’, defined as “a multi-faceted construct that reflects the extent to which people delivering, or, receiving a healthcare intervention consider it to be appropriate, based on anticipated or experienced cognitive and emotional responses to the intervention”.[18,19] In the context of a research program designed to determine the research agenda for

For peer review only

optimising bisphosphonate treatment, the primary aim of this systematic review is to explore the acceptability of different bisphosphonates regimens among patients, and clinicians and managers.

METHODS

We conducted a systematic review and framework synthesis of qualitative studies exploring patient and clinician views and experiences of bisphosphonates. The conduct and reporting of this review followed the Preferred Reporting Items for Systematic Reviews and Meta-Analyses (PRISMA) guidelines (see supplementary material Table 1 for PRISMA checklist). The protocol of the systematic review is registered in PROSPERO [CRD42019143526].

Eligibility

To be eligible for inclusion, studies needed to report on patients', clinicians', academics', and/or manager/payers' experiences and preferences regarding bisphosphonate regimes for adults (≥ 18 years) with osteoporosis. Bisphosphonates needed to be mentioned by name, or there needed to be sufficient information that was specific to bisphosphonate (e.g. reference to the special instructions for use of oral bisphosphonates), to deduce that study findings related to bisphosphonates, as agreed by two clinically experienced authors independently. Papers describing experiences of osteoporosis more generally were included if there were findings relating to bisphosphonate treatment in the study abstract. Studies were only included if they were qualitative in design, or mixed methods with a qualitative component, relevant to a developed country setting and written in English language. Studies were excluded that involved paediatric patients; patients and clinicians receiving/recommending other treatments for osteoporosis; and studies in which bisphosphonates were being used for other indications (e.g. malignancy or Paget's disease).

Search Methods

Systematic searches were conducted in 7 bibliographic databases (MEDLINE, EMBASE, AMED, CINAHLPlus, PsycINFO, ASSIA, and Web of Science [Social Science Citation Index (SSCI) and Conference Proceedings Citation Index- Social

For peer review only

Science & Humanities (CPCI-SSH)] from inception to 15th July 2019. The search strategy utilised database subject headings and text word searching in title, abstract or keywords, combining terms for: 1) bisphosphonates; 2) experiences and preferences; and 3) qualitative research, based on DeJean et al.'s search filter (see supplementary material 2 for full MEDLINE search strategy).[21] Search terms were adapted as appropriate for each database platform.

In addition, grey literature was searched (DART Europe, Open Grey, and NDLTD (National Digital Library of Theses and Dissertations)); the reference lists of all included studies and relevant systematic reviews identified were checked and key studies were citation tracked.

Study Selection

Two-stage screening of articles against eligibility criteria was undertaken. Firstly, titles and abstracts were screened, then full texts. At both stages screening was conducted by sets of two reviewers independently (NC, EC, ZP) and articles were excluded by agreement. Disagreements were resolved through discussion or by third reviewer adjudication.

Data extraction

For each paper data extraction was completed independently by two researchers (ZP and JW or EC and FM). Key findings from the results sections of papers relating to bisphosphonates were extracted; a 'key finding' was defined as any sentence or statement relating to views or experiences of bisphosphonates from the results section of the paper or abstract. Wherever possible, the key finding was extracted as written by the author, with minimal edits only for clarification, description of context or for consistency across papers. For each paper, two authors extracted key findings independently, and subsequently agreed a final list of key findings for each paper. Data were also extracted on participant numbers and demographics, data collection technique, setting and country. Additionally, if available for patients, information was extracted on their bisphosphonate use including type of drug and current status (adherent, non-adherent, decliner).

Quality appraisal

The quality of each study was assessed using the CASP qualitative tool. This tool consists of 10 items split into 3 sections (qualitative suitability, data analysis and

For peer review only - http://bmjopen.bmj.com/site/about/guidelines.xhtml

overall quality). The first two sections consist of items related to qualitative suitability and data analysis, which were evaluated as “yes”, “no”, “unclear” or “partial”. The final section was an assessment based on the overall quality of the paper including the previous items and its relevance to the review objectives, this was rated as “high”, “moderate” or “low”. All papers were quality appraised by two researchers independently (FM, SB, JW). Disagreements were resolved through discussion with a fourth reviewer (ZP).

Synthesis

We used a framework synthesis approach informed by the ‘Best Fit’ model described by Carroll et al.[20] The "best fit" method offered a means to test, reinforce and build on an existing published model, conceived for a different but relevant purpose. This approach was chosen as a published theory was identified from the literature that conceptualised acceptability - the Theoretical Framework of Acceptability (TFA).[18] The TFA is a relatively new framework which was developed to inform the understanding of acceptability of complex interventions, and consists of seven constructs: affective attitudes - the emotions elicited by an intervention; intervention coherence - the extent to which an intervention makes sense; perceived effectiveness - the perceived extent to which intervention will achieve purpose; burden – the amount of effort required to participate in an intervention; self-efficacy – individual’s confidence that they can perform the behaviour(s) required to participate in the intervention; opportunity-costs - the extent to which benefits, profits, or values must be given up to engage in an intervention; and ethicality – the extent to which an intervention has a good fit with an individual’s values. The framework also incorporates temporal perspectives on *anticipated* and *experienced* acceptability at three time points before (prospective), during (experienced) and after (retrospective) experience of an intervention.

The TFA has not previously been used to evaluate drug acceptability. We anticipated the seven constructs of the TFA would be relevant to engagement with drug treatment; for example, burden could relate to treatment burden associated with administrating the drug or side effects. However, one aspect which did not appear to be explicitly conceptualised within the framework was patient beliefs about medicines. Studies across a range of long term conditions, healthcare systems and cultures have consistently shown that engagement with treatment is influenced by

For peer review only

patients' personal evaluation of the medicine in question.[21] Particularly important is how they judge their personal need for treatment relative to their concerns about it. For this reason, we therefore included the Necessity Concerns Framework (NCF) [21], to further explore the TFA domain relating to intervention coherence.

The first author initially conducted inductive open coding on the data extracted, before mapping the codes to a draft framework derived from a priori themes (the domains of the TFA). Authors then met to first discuss the themes and compare findings for each study and the 'fit' to the draft framework. A preliminary synthesis was achieved using tabulation of studies, organising the studies into groups relating to temporal perspectives and research question, and exploring relationships between studies and between groups.

A final coding framework was agreed at a second meeting of authors. A second author (FM) recoded the original key findings, where necessary, to the new framework to ensure all findings were represented. Finally, relationships between themes and TFA and NCF domains were explored by further group discussion. We used the Grades of Recommendation, Assessment, Development, and Evaluation Confidence in the Evidence from Qualitative Reviews (GRADE-CERQual) approach to determine confidence in our synthesised findings.[22]

RESULTS

The literature search identified 2040 unique articles, of which 25 met eligibility criteria (Figure 1), a summary of the studies is shown in Table 1.

The included studies were categorised into three groups: perceptions of osteoporosis generally; [23,24,26,27,31,38,39] healthcare service delivery issues unrelated to osteoporosis (de-prescribing) [35] and inter-professional communication in primary care[34]) and studies specific to osteoporosis treatments. The latter group was further subdivided into: those examining treatment barriers;[16,28,36,37,42,43] adherence;[29–30,44] decision-making;[32–33,40,41,46] or bisphosphonate-related side effects.[25,45] Only one study examining adherence and one examining decision making had research questions which specifically related to bisphosphonates.[30,41]

For peer review only

The majority (23) of studies were conducted in North America or Europe. Eleven studies explored patient views,[23–33] six explored HCPs' views,[34–39] seven had a mixed sample[16,40–45] and two studies interviewed managers.[16,37] No studies included academic or payor participants. Of the 18 papers that included patients, 10 studies described how many of the patients were on anti-osteoporotic medication, however, only two reported the specific type of medication. Only one study reporting patient experience of receiving intravenous bisphosphonate.[31]

For peer review only

Table 1: Summary of included studies

First author and year	Participants[i]	Participant No. (male:female)	Bisphosphonate use and adherence[ii]	Data collection methods	Recruitment setting	Country
**Studies in Group 1: Views of Osteoporosis**						
Besser 2012[23]	Pts	14 (0:14)	AOD unspecified	Interview	One hospital	UK
Jaglal 2003[39]	HCPs<br>Family Physicians (n=32)	32 (12:20)	N/A	Focus group	Primary Care	Canada
Otmar 2012[38]	HCPs<br>GP (n = 14), Practice nurse (n = 2)	16 (11:5)	N/A	Focus group	Primary care	Australia
Sale 2015[24]	Pts	28 (2:26)	19/28 pts on AOD<br>adherent (n=19)<br>declined (n= 4)	Interview	National osteoporosis patient group	Canada
Sale 2010[26]	Pts	24 (6:18)	9/24 pts on AOD,<br>risedronate (n=8),<br>etidronate (n=1)	Focus group	Fracture Clinic	Canada
Weston 2011[27]	Pts	10 (0:10)	AOD unspecified	Interview	Primary Care	UK
Hansen 2017[31]	Pts	15 (0:15)	AOD unspecified<br>adherent (n=12)<br>declined/stopped AOD (n=3)	Interview	Women attending DXA at 2 hospitals	Denmark
**Studies in Group 2: Views of Osteoporosis Treatment (treatment barriers)**						
Alami 2016[42]	Mixed	Pts: 37 (0:37)<br>HCPs: 18 (8:10)	23/47 pts on AOD,<br>adherent (n=19)<br>declined/stopped AOD (n=18)	Focus group	Hospital/community over 5 regions	France
Drew 2016[37]	HCPs<br>Nurse (n = 14), GP (n= 2),<br>Specialists (n =17), Orthopaedic Surgeon (n = 4)<br>Managers (n = 5), DXA technician (n=1)	43 (not given)	N/A	Interview	11 hospitals in one region	UK
Feldstein 2008[16]	Mixed	Pts: 10 (0:10)<br>HCPs: 57 (not given)	AOD unspecified	Interview and focus group	Primary and secondary care	USA
Guzman-Clarke 2007[43]	Mixed	100 (94:6)	24/100 pts on AOD	Focus group	Urban academic Medical Centre	USA
Merle 2019(a)[28]	HCPs (GP)	16 (11:5)	N/A	Interview	Primary Care	France
Merle 2019(b)[36]	Pts	98 (53:45)	AOD Unspecified	Focus group	Recruited from 2 existing research studies and community (medical insurance company)	France
**Studies in Group 2: Views of Osteoporosis Treatment (adherence)**						
Iversen 2011[44]	Mixed	Pts: 32 (2:30)<br>HCPs: 12 (5:7)	AOD unspecified	Focus group	Secondary care	USA
Lau 2008[29]	Pts	37 (0:37)	33/37 pts on AOD, alendronate (n=9), etidronate (n=5),	Focus group	Primary care, secondary care and community pharmacies	Canada

			risedronate (n=19)			
Salter 2014[30]	Pts	30 (0:30)	20/30 pts on AOD<br>adherent (n=19)<br>declined (n=1)<br>stopped AOD (n=10)	Interview	Primary Care	UK
**Studies in Group 2: Views of Osteoporosis Treatment (Decision Making)**						
Mazor 2016[32]	Pts	36 (0:36)	15/36 pts on AOD<br>adherent (n=15)<br>declined (n=10)<br>stopped (n=11)	Telephone Interview	Primary Care	USA
Sale 2011[46]	Pts	24 (6:15)	14/21 pts on AOD	Telephone Interview	Hospital based fracture screening programme	Canada
Swart 2018[40]	Mixed	Pts: 26 (4:22)<br>HCPs: 13 (not given)	10/26 pts on AOD<br>adherent (n=10)<br>declined (n=16)	Interview	Recruited from a fracture prevention study	Netherlands
Scoville 2011[41]	Mixed	Pt: 18 (0:18)<br>HCP: 19 (12:7)	N/A	Videographic	Primary care (osteoporosis choice trial)	USA
Wozniak 2017[33]	Pts	12 (3:9)	7/12 pts on AOD,<br>adherent (n=7)<br>stopped (n=5)	Interview	Recruited from a fracture prevention trial nested in secondary care	Canada
**Studies in Group 2: Views of Osteoporosis Treatment (Bisphosphonate side effects)**						
Sturrock 2019[45]	Mixed	24 (4:19)	13/23 pts on AOD	Interview	Three regions including from secondary care	UK
Sturrock 2017[25]	Pts	17 (7:10)	N/A	Interview	Primary Care	UK
**Studies in Group 3: Non-Specific Osteoporosis Issues**						
Ailabouni 2016[35]	HCPs	10 GPs	N/A	Interview	Primary Care	New Zealand
Sippli 2017[34]	HCPs	28 (6:22)	N/A	Interview	Primary Care	Germany

[i] Pts – patients; HCPs – healthcare professionals
[ii] Where specified. AOD – anti-osteoporosis drug. N/A not applicable

The findings related to quality appraisal are summarised in Table 2. The most common limitations of the included studies were lack of description of author reflexivity, lack of depth of analysis, use of normative statements and relatively small samples or studies conducted in a single site which may limit transferability of the findings. Furthermore, although the characteristics of the sample were generally reasonably described, in order to address our research question, we required information about medication use of participants which was frequently not described.

Using the CASP tool, 12 (48%) studies were scored as high value and the remaining 13 (52%) studies as moderate value. For 8/13 (62%) studies scored as moderate in value, this was because the focus of the paper was less relevant to our research question rather than because of methodological issues.

For peer review only

Table 2. Quality Appraisal

First author and year	*CASP tool Question*[i]										*Comments*[ii]
	**1**	**2**	**3**	**4**	**5**	**6**	**7**	**8**	**9**	**10**	
Group1: Views of Osteoporosis											
Besser 2012[23]	✓	✓	✓	p	✓		✓	p	✓	Moderate	Small sample, no mention of data saturation, limited to 'psychological' factors affecting adherence (discounting other factors by omission) and some use of normative statements
Jaglal 2003[39]	✓	✓	✓	✓	✓	u	✓	✓	✓	Moderate	Few findings relevant to our research question
Otmar 2012[38]	✓	✓	✓	✓	✓		✓	✓	✓	Moderate	Well conducted study, but limited findings relating to bisphosphonates
Sale 2015[24]	✓	✓	✓	✓	✓	u	✓	✓	✓	High	
Sale 2010[26]	✓	✓	✓	p	✓	u	✓	p	✓	Moderate	Small single site study, although data saturation reached. Language does not always appear to match approach (e.g. reporting patient 'inability' to link fractures to osteoporosis suggests prior normative assumptions)
Weston 2011[27]	✓	✓	✓	✓	✓	✓	✓	✓	✓	High	
Group 2: Views of Osteoporosis Treatment											
Alami 2016[42]	✓	✓	✓	✓	✓		✓	✓	✓	High	
Drew 2016[37]	✓	✓	✓	✓	✓	u	✓	✓	✓	High	
Feldstein 2008[16]	✓	✓	✓	✓	✓	u	✓	✓	✓	High	
Guzman-Clarke 2007[43]	✓	✓	✓	✓	✓	u	✓	u	✓	Moderate	Only partially relevant for our review given the focus on a specific population (Glucocorticoid-Induced Osteoporosis)
Merle 2019[28]	✓	✓	✓	p	✓	u	✓	u	✓	Moderate	Small sample (although data saturation reached) without attempt to structure to population and analysis lacks depth to answer our objective relating to bisphosphonate acceptability
Merle 2019[36]	✓	✓	✓	✓	✓	u	✓	✓	✓	Moderate	Limited information relevant to our research question in view of general focus on osteoporosis
Iversen 2011[44]	✓	✓	✓	p	✓		✓	p	✓	Moderate	Single centre study, although data saturation reached, limited information on coding/analysis and no discussion of findings with relevance to wider literature
Lau 2008[29]	✓	✓	✓	✓	✓		✓	✓	✓	High	
Salter 2014[30]	✓	✓	✓	✓	✓		✓	✓	✓	High	

For peer review only

Hansen 2017[31]	✓	✓	✓	✓	✓	✓	✓	✓	✓	High	
Mazor 2016[32]	✓	✓	✓	✓	✓	u	✓	u	✓	Moderate	Good relevance, single site. Descriptive approach without critical reflexivity or discussion of prior assumptions
Sale 2011[46]	✓	✓	✓	✓	✓	u	✓	✓	✓	High	
Swart 2018[40]	✓	✓	✓	✓	✓	✓	✓	✓	✓	High	
Scoville 2011[41]	✓	✓	✓	✓	✓	u	✓	✓	✓	Moderate	Well conducted videographic study, but data coded against deductive categories of reasons to reject treatment, so limited potential to inform our objective about acceptability
Wozniak 2017[33]	✓	✓	✓	✓	✓	u	✓	✓	✓	High	
Sturrock 2019[45]	✓	✓	✓	✓	✓	u	✓	✓	✓	High	
Sturrock 2017[25]	✓	✓	✓	✓	✓		✓	✓	✓	Moderate	Aim only partially relevant to study question
Group 3: Non-specific Osteoporosis Issues											
Ailabouni 2016[35]	✓	✓	✓	p	✓	✓	✓	✓	✓	Moderate	Relatively small (10 respondents) study, although data saturation reached. Only partial relevant for current review with brief coverage of GPs views on discontinuing bisphosphonates in light of multimorbidities
Sippli 2017[34]	✓	✓	✓	✓	✓		✓	✓	✓	Moderate	Limited findings related to our research question

[i]CASP Quality assessment questions; 1, Was there a clear statement of the aims of the research? 2, Is a qualitative methodology appropriate? 3, Was the research design appropriate to address the aims of the research? 4, Was the recruitment strategy appropriate to the aims of the research? 5, Was the data collected in a way that addressed the research issue? 6, Has the relationship between researcher and participants been adequately considered? 7, Have ethical issues been taken into consideration? 8, Was the data analysis sufficiently rigorous? 9, Is there a clear statement of findings? 10, Value of study and relevance to review objectives. ✓ = Yes, u = Unsure, p = partial, blank = No.

[ii] Comments only made for those ranked Moderate or Low.

Fifteen individual sub-themes were identified which mapped to the seven domains of the TFA. Key findings relating to ethicality related to conflict between bisphosphonates and participants' values and were usually discussed as part of sense making. For this reason, issues relating to 'ethicality' were considered as part of 'intervention coherence', leaving six main themes, as shown schematically in Figure 2. Although it was possible to distinguish between two temporal perspectives, related to anticipated and experienced acceptability within most domains (with the exception of self-efficacy) the majority of anticipated acceptability findings related to intervention coherence.

The findings of the review are discussed below with GRADE-CERQual ratings of confidence in Table 3 and illustrative key findings for each theme/subtheme shown in Supplementary material 3. Subthemes are identified in the text in italics.

For peer review only

Table 3: GRADE-CERQual Summary of qualitative findings table

Review Finding [and contributing studies]	Methodological limitations	Coherence	Adequacy	Relevance	*CERQual Confidence assessment*
	Concerns				
**Intervention coherence:** Both before starting, and during treatment, patients considered the perceived need or necessity for bisphosphonates based on their views of osteoporosis, including its seriousness and controllability, symptoms and their perception of their own health. Perceived need was weighed up against concerns about medication, including suspicion of drugs in general and specific concerns about bisphosphonate safety by both patients and HCPs. HCPs sometimes used principles of ethicality to support perceptions of low necessity and their reluctance to prescribe. The decision process of balancing necessity against concerns, was influenced by the doctor-patient relationship and wider societal influences including friends, family and the general media. This process influenced whether HCPs reported recommending bisphosphonates. For patients, the decision process could be explicit or tacit, was revisited over time and influenced both whether they initiated treatment and subsequently adhered. [16,23,33,35,36,38–44,24,45,46,257,26,28–32]	*Minor*<br><br>12/22 papers rated moderate value due to sample size, depth of analysis or lack of reflexivity[i]	*None or very minor*<br><br>The finding reflects the complexity and variation of the data, and these influences on sense making are well supported by details in the underlying studies	*None or very minor*<br><br>22 papers contributed to this finding, and although some gave little detail, in-depth insights were reported in 10 papers and information was consistent across studies	*Minor*<br><br>Spread of studies from primary and secondary care and range of countries. Uncertainties remain about sense making related patients taking intravenous bisphosphonates and influence of gender	HIGH
**Perceived effectiveness** Both patients and HCPs expressed doubt or uncertainty about the mechanism of effectiveness of bisphosphonates and expressed a range of treatment expectations including strengthening bone - improving bone density, preventing worsening of osteoporosis - maintaining bone density and/or total fracture prevention. Patients wanted proof or evidence of effectiveness through more structured monitoring and follow-up, and were disincentivised to continue treatment in the absence of evidence of perceived effectiveness. [16,23,24,30,32,37,39–42,44]	*Minor*<br><br>7/15 papers rated moderate value, mostly (4/7) due to limited relevant content. Methodological concerns relate to depth of analysis or lack of reflexivity[i]	*None or very minor*<br><br>The finding reflects the complexity and variation of the data, and these issues are supported by details in the underlying studies	*None or very minor*<br><br>15 papers contributed to this finding. Some gave little detail, but in-depth insights were reported in 6 papers and information was consistent	*Minor*<br><br>Spread of studies from primary and secondary care and range of countries. Uncertainties remain about perceived effectiveness of intravenous bisphosphonates	HIGH
**Self-efficacy** Measures to help patients integrate medication taking into daily routines (supporting routinisation), and the provision of information and support, enhanced their feeling of having control over their health and confidence to adhere to bisphosphonates. Clinician reported barriers to supporting adherence related to perceptions of their knowledge and attitudes, with several knowledge gaps and uncertainties reported, and the perception that osteoporosis was not a priority. Finally, service level barriers which impaired clinicians' self-efficacy in recommending and managing patients on bisphosphonates, included uncertainty	*Minor*<br><br>7/15 papers rated moderate value, mostly (4/7) due to limited relevant content. Methodological concerns relate to	*None or very minor*<br><br>The finding reflects the complexity and variation of the data, and these issues are supported by details in the underlying studies	*None or very minor*<br><br>17 papers contributed to this finding. Some gave little detail, but in-depth insights were reported in 5 papers and	*Minor*<br><br>Spread of studies from primary and secondary care and range of countries. Uncertainties remain about self-efficacy relating to	HIGH

For peer review only

about professional roles and responsibilities, capacity, access to intravenous drugs and communication and IT systems. [16,24,37–39,42–45,25,27–31,34,35]	depth of analysis or sample size[i]		information was consistent.	intravenous bisphosphonates	
**Affective attitudes:** The emotions elicited by bisphosphonates were closely related to intervention coherence. Bisphosphonates were associated predominantly with negative emotions of fear (of side effects) and annoyance (with special instructions); however positive emotions of reassurance and hope were noted in two studies, linked to the anticipated protection that bisphosphonates could incur. [16,23,27,29–32,42]	*Minor* 2/8 papers rated moderate value due to depth of analysis or lack of reflexivity[i]	*None or very minor* The finding reflects the data, supported by details in the underlying studies	*Moderate* Reports of affective attitude were mostly descriptive with little depth	*Moderate* Uncertainties remain about affective attitudes to injectable bisphosphonates received in hospital	MODERATE
**Burden:** The burden or effort of oral bisphosphonates was described mostly relating to the special instructions to take oral bisphosphonates or experienced side effects, although costs incurred were also a potential source of burden. [16,23,45,27–31,40,41,44]	*Minor* 4/11 papers rated moderate value due to sample size, depth of analysis[i]	*None or very minor* The finding reflects the data, and these aspects of burden are supported by details in the underlying studies	*Moderate* Reports mostly descriptive with little depth and a possible focus on presence of burden (side effects) rather than absence	*Moderate* Uncertainties remain about burden of indirect costs (travel, dental checks) & burden due to intravenous bisphosphonates	MODERATE
**Opportunity costs** Circumstances where competing priorities challenged adherence or initiation of bisphosphonates were described relating to co-morbid conditions. The presence of comorbid conditions were described as resulting in less time to support discussion about bisphosphonates in consultations and, result in recommendation of, and adherence to, bisphosphonates being given relative low priority. [16,25,46,28,30,31,33,36,39,40,45]	*None or very minor* 4/11 papers rated moderate value, but this was mostly (n=3) due to limited relevant content rather than methodological concerns.	*Moderate* No discussion of the alternative explanation that having co-morbid conditions may facilitate bisphosphonate acceptability	*Moderate* Reports were limited, lacked depth & 3 papers contained little content relevant to the research question	*Moderate* No information about values, benefits that have to be given up to partake in intravenous bisphosphonates, which are likely to be different & likely limited sampling of patients with complex health needs	LOW

[i] Concerns considered minor because of the methodological strength of the other papers in this domain, & low likelihood that reflexivity would affect finding

For peer review only

Intervention Coherence (high confidence)

Both before starting, and during treatment, patients considered the perceived need or *necessity* for bisphosphonates based on their views of osteoporosis, including its seriousness and controllability, symptoms and their *perception of their own health.* Perceived need was weighed up against *concerns* about medication, including suspicion of drugs in general and specific concerns about bisphosphonate safety, by both patients and HCPs. HCPs sometimes used principles of *ethicality* to support perceptions of low necessity and their reluctance to prescribe. The *decision process* of balancing necessity against concerns, was influenced by the doctor-patient relationship and wider societal influences including friends, family and the general media. This process influenced whether HCPs reported recommending bisphosphonates. For patients, the decision process could be explicit or tacit, was revisited over time and influenced both whether they initiated treatment and subsequently adhered.

Perceived Effectiveness (high confidence)

Both patients and HCPs expressed doubt or uncertainty about the *mechanism of effectiveness* of bisphosphonates and expressed a range of treatment expectations including strengthening bone - improving bone density, preventing worsening of osteoporosis - maintaining bone density and/or total fracture prevention. Patients wanted proof or evidence of effectiveness through more structured *monitoring and follow-up,* and were disincentivised to continue treatment in the absence of evidence of perceived effectiveness.

Self-efficacy (high confidence)

Measures to help patients integrate medication taking into daily routines (*supporting routinisation*), and the provision of information and support, enhanced their feeling of having control over their health and confidence to adhere to bisphosphonates. Clinician reported barriers to supporting adherence related to perceptions of their *knowledge and attitudes,* with several knowledge gaps and uncertainties reported, and the perception that osteoporosis was not a priority. Finally, s*ervice level barriers* which impaired clinicians' self-efficacy in recommending and managing patients on

For peer review only

bisphosphonates, included uncertainty about professional roles and responsibilities, capacity, access to intravenous drugs and communication and IT systems.

Affective attitudes (moderate confidence)

The *emotions* elicited by bisphosphonates were closely related to intervention coherence. Bisphosphonates were associated predominantly with negative emotions of fear (of side effects) and annoyance (with special instructions); however positive emotions of reassurance and hope were noted in two studies, linked to the anticipated protection that bisphosphonates could incur.

Burden (moderate confidence)

The burden or effort of oral bisphosphonates was described mostly relating to the *special instructions* to take oral bisphosphonates or experienced *side effects*, although *costs* incurred were also a potential source of burden. Only one study included the experience of a patient on an intravenous bisphosphonate, this patient described low treatment burden as she only had to go once a year, and felt no side effects.[31]

Opportunity costs (low confidence)

There were few descriptions of 'benefits, profits, or values' being given up to take bisphosphonates. However, circumstances where competing priorities challenged adherence or initiation of bisphosphonates were described relating to *co-morbid conditions*. The presence of comorbid conditions was described as resulting in less time to support discussion about bisphosphonates in consultations and, result in recommendation of, and adherence to, bisphosphonates being given relative low priority.

For peer review only

DISCUSSION

This systematic review has used the lens of acceptability to understand perceptions of bisphosphonates and the problem of poor adherence. We have identified, with high confidence, how patients and HCPs make sense (coherence) of bisphosphonates by balancing perceptions of need against concerns, how uncertainty prevails about perceived effectiveness of bisphosphonates and how a number of individual and service factors have potential to increase self-efficacy in recommending and adhering to bisphosphonates. We identified with moderate confidence, that bisphosphonate taking induces fear, but has the potential to engender reassurance, and that both the side effects and special instructions for taking oral bisphosphonates can be a source of treatment burden. Finally, we identified with low confidence that multi-morbidity plays a role in people's perception of bisphosphonate acceptability.

To our knowledge, this is the first use of the Theoretical Framework for Acceptability, originally developed to evaluate acceptability of complex interventions, to evaluate the acceptability of medication. We explored the utility of the TFA from two perspectives, as an explanatory model for both patient and clinician acceptability and engagement. The TFA was useful for understanding and combining patient and clinician viewpoints; however, there was considerable overlap between domains; perceived efficacy, affective attitudes and self-efficacy beliefs are all likely to impinge on sense-making, or intervention coherence. The TFA alone does not provide a comprehensive framework for understanding patient acceptability or engagement with medicines, and of course it was not intended to do so. The sense-making aspect of the framework appeared pivotal, and the explanatory value of the framework was enhanced by the incorporation of the NCF to operationalise key engagement related beliefs. In the context of bisphosphonates, concern and associated fears predominate among patients, and perceived need may be underestimated if the consequences of osteoporosis and fragility fractures are not explained. In our findings, sense making was dynamic. Patients re-evaluated perceptions of bisphosphonates over time, expressing uncertainty relating to what represents successful treatment and citing perceived lack of effectiveness being cited as reason to discontinue. This is likely to be a particular problem for bisphosphonates, as opposed to other drugs commonly taken for prevention such as

For peer review only

statins and anti-hypertensive, where measures of feedback and effectiveness are more readily available.

The NICE guidelines for medicines adherence emphasises the need to take into account perceptions (e.g. necessity beliefs and concerns) and practicalities (e.g. capability and resources) that will affect individuals' motivation and ability to start and continue with treatment.[47] However, interventions designed to improve bisphosphonate adherence are often designed to 'educate' or persuade the patient of importance and are often not targeted to eliciting or addressing health beliefs, or informed by underpinning mechanisms of change.[3] There is therefore a need to ensure that any further design of interventions - to promote bisphosphonate adherence - draws on more comprehensive theoretical models of patient engagement with health conditions and medicines such as the Extended Common Sense Model.[48] Specifically, our findings suggest a need for clinicians to support patients to understand the need for treatment, to allay concerns where possible and to define what constitutes successful bisphosphonate treatment. Furthermore, clinicians need to support patients evaluate the advantages and disadvantages over time, given the dynamic nature of these decision processes.[48]

It is clear from our findings that clinicians also have necessity-concern dilemmas relating to bisphosphonates. A number of studies reported clinicians themselves perceiving low patient need, high concerns and perceptions treatment was not practical. This is perhaps in contrast with a previous quantitative study in asthma which demonstrated that clinicians held stronger positive beliefs about medicines than patients.[49] It is unclear to what extent the perceptions in our findings were generalisations or applied in specific circumstances, or to what extent these views were negotiated on an individual basis in discussion with patients. Problems may arise in the consultation if clinicians assume patients share their views and then may be less likely to explore patient perceptions of need or concerns. Furthermore, the limitations of interviewing HCPs are well documented; the accounts presented in an interview may not represent clinician underlying beliefs or behaviours meaning that observational methods may be more appropriate to fully understand clinical decisional making.[50] Given the clinician has a pivotal role in sense making, interventions are also likely needed to address clinician knowledge, attitudes and beliefs. By including the views of clinicians and managers we have also identified a

For peer review only

range of service level barriers to promoting bisphosphonate adherence relating to lack of clarity about professional roles, both across primary and secondary care, and within primary care, use of IT systems and access to intravenous treatments.

A strength of this review is the comprehensive search, use of underpinning theoretical framework, the inclusion of clinician views in addition to patients, and the use of the GRADE-CERQual to give confidence in our findings which has facilitated a clear identification of where further research is needed. Areas where we have identified moderate or low confidence are in need of further research and specifically relate to the influence of multi-morbidity on sense making, burden and self-efficacy in bisphosphonate users, the extent to which intravenous bisphosphonates may overcome issues related to treatment burden and self-efficacy, and the impact of bisphosphonates on affective attitudes and emotions. Furthermore, we have identified gaps in our understanding of how clinicians make decisions in practice, and how views of bisphosphonates may be influenced by gender. Given that many osteoporosis drugs have a different evidence base and licensing arrangements in men this is an area in need of further study.

The main limitation of this study relates to the lack of clarity in many of the included studies in the results sections about which osteoporosis treatments or bisphosphonates were being referred to, meaning that in some cases we may have over-interpreted findings relating to bisphosphonates that were about other osteoporosis drugs. However, all of our review findings were identified from comparison of data from several studies, and as bisphosphonates represent the mainstay of osteoporosis treatment, we consider that over-interpretation is unlikely. As there was frequently little detail about medication participants were taking or referring to, it is also possible that we have missed relevant studies. Only two studies reported the views of managers but unfortunately neither of these studies distinguished professional roles in the presentation of results, so a further need exists to explore perceptions of this group, and perceptions of payors and academics.

For peer review only

CONCLUSIONS

In summary, using the lens of acceptability, we have identified the factors that influence how patients and clinicians make sense of bisphosphonates, described the experience of bisphosphonate taking in terms of burden and factors that both facilitate and hinder confidence in taking, and prescribing and monitoring bisphosphonates. Our findings demonstrate the need for a theoretically informed, whole-system approach' to enable clinicians and patients to get the best from bisphosphonate treatment. Patients need comprehensive support that takes account of the perceptions (e.g. treatment necessity beliefs and concerns) and practicalities (e.g. capability and resources) that influence their motivation and ability to start and continue with treatment. Clinicians need to moderate patient expectations and clarify what constitutes bisphosphonate treatment success. Finally, further research is needed to explore perspectives of managers, patients receiving intravenous bisphosphonates, men receiving bisphosphonates and the use of bisphosphonates in the context of multi-morbidity.

ACKNOWLEDGEMENTS/FUNDING

This study is funded by the National Institute for Health Research (NIHR), [HTA NIHR127550]. ZP is funded by the NIHR, Clinician Scientist Award (CS-2018-18-ST2-010)/NIHR Academy. CJ is part funded by the NIHR Applied Research Collaboration West Midlands. The views expressed are those of the author(s) and not necessarily those of the National Health Service, the NIHR, or the Department of Health & Social Care.

CONFLICTS OF INTEREST

None declared

For peer review only

REFERENCES

1 Cooper C, Campion G, Melton LJ. Hip fractures in the elderly: A world-wide projection. *Osteoporos Int* 1992;2:285-9. doi:10.1007/BF01623184

2 National Osteoporosis Society. NHS RightCare scenario: The variation between sub-optimal and optimal pathways. *Rep* 2017:1–16.

3 Hiligsmann, M., Cornelissen, D., Vrijens, B. et al. Determinants, consequences and potential solutions to poor adherence to anti-osteoporosis treatment: results of an expert group meeting organized by the European Society for Clinical and Economic Aspects of Osteoporosis, Osteoarthritis and Musculoskeletal Diseases (ESCEO) and the International Osteoporosis Foundation (IOF). *Osteoporos Int* 2019;30:2155–65. https://doi.org/10.1007/s00198-019-05104-5

4 Khosla S, Shane E. A Crisis in the Treatment of Osteoporosis. *J Bone Miner Res* 2016;31:1485-7. doi:10.1002/jbmr.2888

5 May CR, Montori VM, Mair FS. We need minimally disruptive medicine. *BMJ* 2009;339:2830. doi:10.1136/bmj.b2803

6 National Institute for Health Care Excellence. Bisphosphonates for treating osteoporosis, Technology appraisal guidance (TA46) *NICE Guidance* 2017

7 Jha S, Wang Z, Laucis N, et al. Trends in Media Reports, Oral Bisphosphonate Prescriptions, and Hip Fractures 1996-2012: An Ecological Analysis. *J Bone Miner Res* 2015;30:2179-87 doi:10.1002/jbmr.2565

8 Recknor C, Czerwinski E, Bone HG, et al. Denosumab compared with ibandronate in postmenopausal women previously treated with bisphosphonate therapy: A Randomized Open-Label Trial. *Obstet Gynecol* 2013;121:1291-9. doi:10.1097/AOG.0b013e318291718c

9 Akarirmak Ü, Koçyiğit H, Eskiyurt N, et al. Influence of patient training on persistence, compliance, and tolerability of different dosing frequency regimens of bisphosphonate therapy: An observational study in Turkish patients with postmenopausal osteoporosis. *Acta Orthop Traumatol Turc 2016;50;415-23.* doi:10.1590/S0080-62342016000500008

For peer review only

10 Kishimoto H, Maehara M. Compliance and persistence with daily, weekly, and monthly bisphosphonates for osteoporosis in Japan: analysis of data from the CISA. *Arch Osteoporos* 2015;10:27. doi:10.1007/s11657-015-0231-6

11 Durden E, Pinto L, Lopez-Gonzalez L, et al. Two-year persistence and compliance with osteoporosis therapies among postmenopausal women in a commercially insured population in the United States. *Arch Osteoporos* 2017;12:22. doi:10.1007/s11657-017-0316-5

12 Saini SD, Schoenfeld P, Kaulback K, et al. Effect of medication dosing frequency on adherence in chronic diseases. *Am J Manag Care* 2009;15:22-33.

13 Hiligsmann M, Bours SPG, Boonen A. A Review of Patient Preferences for Osteoporosis Drug Treatment. *Curr Rheumatol Rep* 2015;17:61. doi:10.1007/s11926-015-0533-0

14 de Bekker-Grob EW, Essink-Bot ML, Meerding WJ, et al. Patients' preferences for osteoporosis drug treatment: A discrete choice experiment. *Osteoporos Int* 2008;19:1029-37. doi:10.1007/s00198-007-0535-5

15 Alten R, Krüger K, Rellecke J, et al. Examining patient preferences in the treatment of rheumatoid arthritis using a discrete-choice approach. *Patient Prefer Adherence* 2016;10:2217. doi:10.2147/PPA.S117774

16 Feldstein AC, Schneider J, Smith DH, et al. Harnessing stakeholder perspectives to improve the care of osteoporosis after a fracture. *Osteoporos Int* 2008;19:1527-40. doi:10.1007/s00198-008-0605-3

17 Bliuc D, Eisman JA, Center JR. A randomized study of two different information-based interventions on the management of osteoporosis in minimal and moderate trauma fractures. *Osteoporos Int* 2006;19:1309-17. doi:10.1007/s00198-006-0078-1

18 Sekhon M, Cartwright M, Francis JJ. Acceptability of healthcare interventions: An overview of reviews and development of a theoretical framework. *BMC Health Serv Res* 2017;17:88. doi:10.1186/s12913-017-2031-8

19 Dejean D, Giacomini M, Simeonov D, et al. Finding Qualitative Research

For peer review only

Evidence for Health Technology Assessment. *Qual Health Res* 2016;26:1307-17. doi:10.1177/1049732316644429

20 Carroll C, Booth A, Cooper K. A worked example of 'best fit' framework synthesis: A systematic review of views concerning the taking of some potential chemopreventive agents. *BMC Med Res Methodol* 2011:11:29. doi:10.1186/1471-2288-11-29

21 Horne R, Chapman SCE, Parham R, et al. Understanding patients' adherence-related Beliefs about Medicines prescribed for long-term conditions: A meta-analytic review of the Necessity-Concerns Framework. *PLoS One* 2013;8:e80633. doi:10.1371/journal.pone.0080633

22 Lewin S, Booth A, Glenton C, et al. Applying GRADE-CERQual to qualitative evidence synthesis findings: Introduction to the series. *Implement Sci* 2018;2. doi:10.1186/s13012-017-0688-3

23 Besser SJ, Anderson JE, Weinman J. How do osteoporosis patients perceive their illness and treatment? Implications for clinical practice. *Arch Osteoporos* 2012;7:115-24 doi:10.1007/s11657-012-0089-9

24 Sale JEM, Hawker G, Cameron C, et al. Perceived messages about bone health after a fracture are not consistent across healthcare providers. *Rheumatol Int* 2015;35:91-130. doi:10.1007/s00296-014-3079-y

25 Sturrock A, Preshaw P, Hayes C, et al. Attitudes and perceptions of GPs and community pharmacists towards their role in the prevention of bisphosphonate-related osteonecrosis of the jaw: A qualitative study in the North East of England. *BMJ Open* 2017;7:e016047. doi:10.1136/bmjopen-2017-016047

26 Sale JEM, Beaton D, Sujic R, et al. 'If it was osteoporosis, i would have really hurt myself.' Ambiguity about osteoporosis and osteoporosis care despite a screening programme to educate fragility fracture patients. *J Eval Clin Pract* 2010;16:590-6. doi:10.1111/j.1365-2753.2009.01176.x

27 Weston JM, Norris E V., Clark EM. The invisible disease: Making sense of an osteoporosis diagnosis in older age. *Qual Health Res* 2011;21:192-704. doi:10.1177/1049732311416825

For peer review only

28 Merle B, Haesebaert J, Bedouet A, et al. Osteoporosis prevention: Where are the barriers to improvement in French general practitioners? A qualitative study. *PLoS One* 2019;14:e0219681.

29 Lau E, Papaioannou A, Dolovich L, et al. Patients' adherence to osteoporosis therapy: Exploring the perceptions of postmenopausal women. *Can Fam Physician* 2008;54:394-402.

30 Salter C, McDaid L, Bhattacharya D, et al. Abandoned Acid? Understanding adherence to bisphosphonate medications for the prevention of osteoporosis among older women: A qualitative longitudinal study. *PLoS One* 2014;9. doi:10.1371/journal.pone.0083552

31 Hansen CA, Abrahamsen B, Konradsen H, et al. Women's lived experiences of learning to live with osteoporosis: A longitudinal qualitative study. *BMC Womens Health* 2017;17:17. doi:10.1186/s12905-017-0377-z

32 Mazor KM, Velten S, Andrade SE, et al. Older womens views about prescription osteoporosis medication: A cross-sectional, qualitative study. *Drugs and Aging* 2010;27:999-1008. doi:10.2165/11584790-000000000-00000

33 Wozniak LA, Johnson JA, McAlister FA, et al. Understanding fragility fracture patients' decision-making process regarding bisphosphonate treatment. *Osteoporos Int* 2017;28:219-29. doi:10.1007/s00198-016-3693-5

34 Sippli K, Rieger MA, Huettig F. GPs' and dentists' experiences and expectations of interprofessional collaboration: Findings from a qualitative study in Germany. *BMC Health Serv Res* 2017;17:179. doi:10.1186/s12913-017-2116-4

35 Ailabouni NJ, Nishtala PS, Mangin D, et al. General practitioners' insight into deprescribing for the multimorbid older individual: A qualitative study. *Int J Clin Pract* 2016;70:261-76. doi:10.1111/ijcp.12780

36 Merle B, Dupraz C, Haesebaert J, et al. Osteoporosis prevention: where are the barriers to improvement in a French general population? A qualitative study. *Osteoporos Int* 2019;30:177–85. doi:10.1007/s00198-018-4720-5

37 Drew S, Judge A, Cooper C, et al. Secondary prevention of fractures after hip

For peer review only

fracture: a qualitative study of effective service delivery. *Osteoporos Int* 2016;27:1719-27. doi:10.1007/s00198-015-3452-z

38 Otmar R, Reventlow SD, Nicholson GC, et al. General medical practitioners' knowledge and beliefs about osteoporosis and its investigation and management. *Arch Osteoporos* 2012;7:104-14. doi:10.1007/s11657-012-0088-x

39 Jaglal SB, Carroll J, Hawker G, et al. How are family physicians managing osteoporosis?: Qualitative study of their experiences and educational needs. *Can Fam Physician* 2003;49:462-8.

40 Swart KMA, Van Vilsteren M, Van Hout W, et al. Factors related to intentional non-initiation of bisphosphonate treatment in patients with a high fracture risk in primary care: A qualitative study. *BMC Fam Pract* 2018;19:141. doi:10.1186/s12875-018-0828-0

41 Scoville EA, de Leon Lovaton PP, Shah ND, et al. Why do women reject bisphosphonates for osteoporosis? a videographic study. *PLoS One* 2011;6. doi:10.1371/journal.pone.0018468

42 Alami S, Hervouet L, Poiraudeau S, et al. Barriers to effective postmenopausal osteoporosis treatment: A qualitative study of patients' and practitioners' views. *PLoS One* 2016;11. doi:10.1371/journal.pone.0158365

43 Guzman-Clark JRS, Fang MA, Sehl ME, et al. Barriers in the management of glucocorticoid-induced osteoporosis. *Arthritis Care Res* 2007;140-6. doi:10.1002/art.22462

44 Iversen MD, Vora RR, Servi A, et al. Factors affecting adherence to osteoporosis medications: A focus group approach examining viewpoints of patients and providers. *J. Geriatr. Phys. Ther* 2011;34:72. doi:10.1097/JPT.0b013e3181ff03b4

45 Sturrock A, Preshaw PM, Hayes C, et al. Perceptions and attitudes of patients towards medication-related osteonecrosis of the jaw (MRONJ): A qualitative study in England. *BMJ Open* 2019;9:e024376. doi:10.1136/bmjopen-2018-024376

For peer review only

46 Sale JEM, Gignac MA, Hawker G, et al. Decision to take osteoporosis medication in patients who have had a fracture and are 'high' risk for future fracture: A qualitative study. *BMC Musculoskelet Disord* 2011;12:92. doi:10.1186/1471-2474-12-92

47 Nunes V, Neilson J, O'flynn N, et al. Medicines adherence: involving patients in decisions about prescribed medicines and supporting adherence (CG76). *NICE Guidance* 2009.

48 Horne R, Cooper V, Wileman V, et al. Supporting Adherence to Medicines for Long-Term Conditions. *Eur Psychol* 2019. doi: 10.1027/1016-9040/a000353

49 Driesenaar JA, De Smet PA, van Hulten R, et al. Beliefs about inhaled corticosteroids: comparison of community pharmacists, pharmacy technicians and patients with asthma. *J. Asthma* 2016;53:1051-8.

50 Checkland K, Harrison S, Marshall M. Is the metaphor of 'barriers to change' useful in understanding implementation? Evidence from general medical practice. *J Heal Serv Res Policy* 2007;12:95-00. doi:10.1258/135581907780279657

For peer review only

Figure legends

Figure 1. Prisma Diagram

Figure 2. Identified themes and subthemes mapped to the Theoretical Framework of Acceptability (TFA)

For peer review only

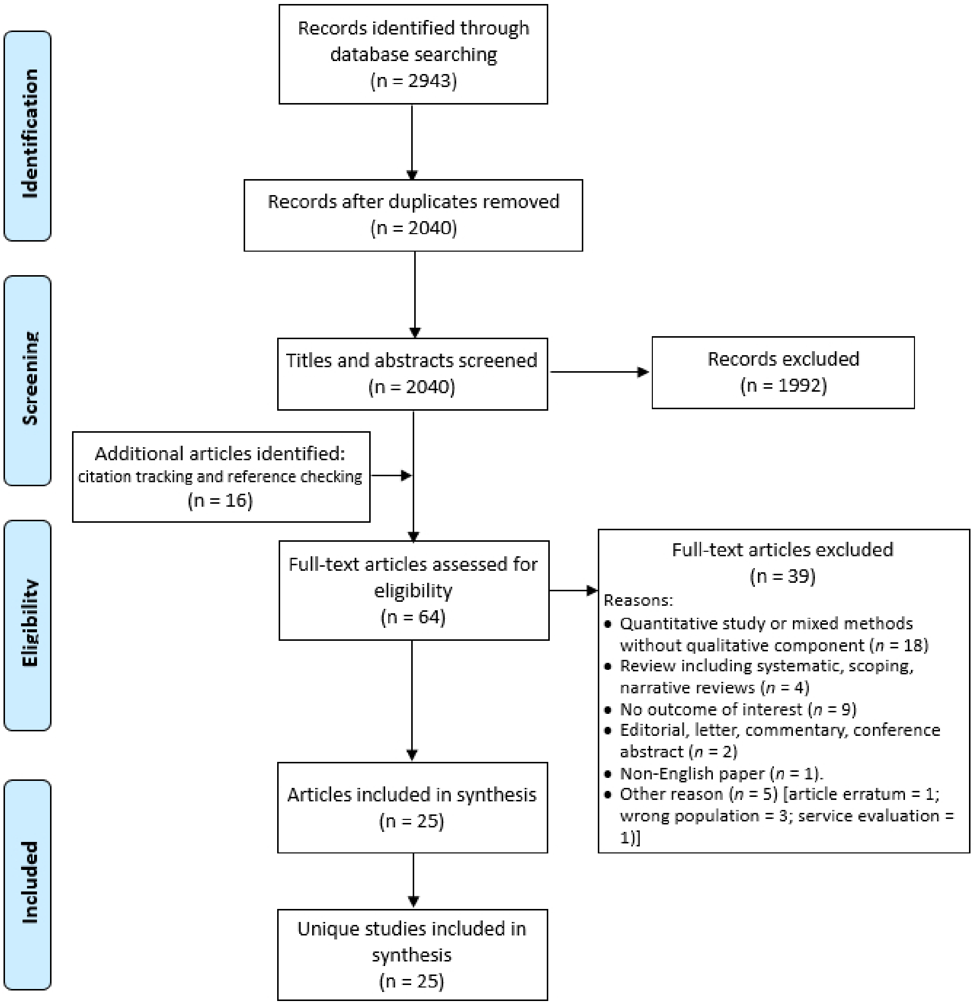

Prisma Diagram

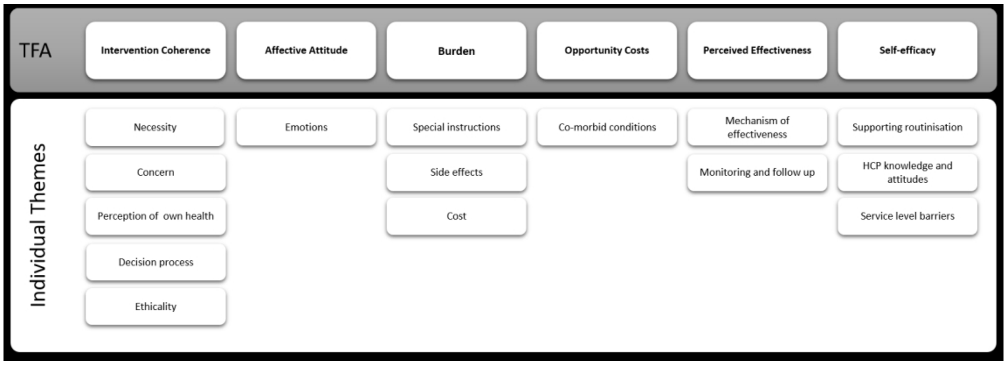

Identified themes and subthemes mapped to the Theoretical Framework of Acceptability (TFA)

Supplementary Material

Table 1. PRISMA 2009 Checklist

Section/topic	#	Checklist item	Reported on page #
**TITLE**			
Title	1	Identify the report as a systematic review, meta-analysis, or both.	1
**ABSTRACT**			
Structured summary	2	Provide a structured summary including, as applicable: background; objectives; data sources; study eligibility criteria, participants, and interventions; study appraisal and synthesis methods; results; limitations; conclusions and implications of key findings; systematic review registration number.	3
**INTRODUCTION**			
Rationale	3	Describe the rationale for the review in the context of what is already known.	4-5
Objectives	4	Provide an explicit statement of questions being addressed with reference to participants, interventions, comparisons, outcomes, and study design (PICOS).	5
**METHODS**			
Protocol and registration	5	Indicate if a review protocol exists, if and where it can be accessed (e.g., Web address), and, if available, provide registration information including registration number.	5
Eligibility criteria	6	Specify study characteristics (e.g., PICOS, length of follow-up) and report characteristics (e.g., years considered, language, publication status) used as criteria for eligibility, giving rationale.	5-6
Information sources	7	Describe all information sources (e.g., databases with dates of coverage, contact with study authors to identify additional studies) in the search and date last searched.	6
Search	8	Present full electronic search strategy for at least one database, including any limits used, such that it could be repeated.	Supplementary material
Study selection	9	State the process for selecting studies (i.e., screening, eligibility, included in systematic review, and, if applicable, included in the meta-analysis).	6-7
Data collection process	10	Describe method of data extraction from reports (e.g., piloted forms, independently, in duplicate) and any processes for obtaining and confirming data from investigators.	7

For peer review only

Data items	11	List and define all variables for which data were sought (e.g., PICOS, funding sources) and any assumptions and simplifications made.	7
Risk of bias in individual studies	12	Describe methods used for assessing risk of bias of individual studies (including specification of whether this was done at the study or outcome level), and how this information is to be used in any data synthesis.	7
Summary measures	13	State the principal summary measures (e.g., risk ratio, difference in means).	7
Synthesis of results	14	Describe the methods of handling data and combining results of studies, if done, including measures of consistency (e.g., I^2) for each meta-analysis.	7-9
Risk of bias across studies	15	Specify any assessment of risk of bias that may affect the cumulative evidence (e.g., publication bias, selective reporting within studies).	9
Additional analyses	16	Describe methods of additional analyses (e.g., sensitivity or subgroup analyses, meta-regression), if done, indicating which were pre-specified.	9
**RESULTS**			
Study selection	17	Give numbers of studies screened, assessed for eligibility, and included in the review, with reasons for exclusions at each stage, ideally with a flow diagram.	9
Study characteristics	18	For each study, present characteristics for which data were extracted (e.g., study size, PICOS, follow-up period) and provide the citations.	11-12
Risk of bias within studies	19	Present data on risk of bias of each study and, if available, any outcome level assessment (see item 12).	12-14
Results of individual studies	20	For all outcomes considered (benefits or harms), present, for each study: (a) simple summary data for each intervention group (b) effect estimates and confidence intervals, ideally with a forest plot.	12-14
Synthesis of results	21	Present results of each meta-analysis done, including confidence intervals and measures of consistency.	16-19
Risk of bias across studies	22	Present results of any assessment of risk of bias across studies (see Item 15).	16
Additional analysis	23	Give results of additional analyses, if done (e.g., sensitivity or subgroup analyses, meta-regression [see Item 16]).	16-17
**DISCUSSION**			
Summary of evidence	24	Summarize the main findings including the strength of evidence for each main outcome; consider their relevance to key groups (e.g., healthcare providers, users, and policy makers).	20-21
Limitations	25	Discuss limitations at study and outcome level (e.g., risk of bias), and at review-level (e.g., incomplete retrieval of identified research, reporting bias).	22

For peer review only

Conclusions	26	Provide a general interpretation of the results in the context of other evidence, and implications for future research.	22-23
**FUNDING**			
Funding	27	Describe sources of funding for the systematic review and other support (e.g., supply of data); role of funders for the systematic review.	23

For peer review only

Supplementary material 2. OVID MEDLINE Search strategy

For Ovid: The following table is an explanation of the symbols used in the search strategy below.

/	indicates an index term (MeSH/EMTREE heading).
exp	before an index term indicates that all subheadings were selected.
af.	Indicates a search for a term in all fields.
.ti,ab,kf.	indicates a search for a term in title/abstract/word(s) in keyword [MEDLINE].
mp.	indicates a search for a term in 'multi-purpose' fields, including the title, abstract, floating sub-heading word, keyword heading word, subject heading word.
tw.	Indicates a search for a term in title and abstract.
$	at the end of a term indicates that this term has been truncated.
?	optional wild card character replaces zero or one character within a word or at the end of a word
adj	indicates a search for tw.o terms where they appear adjacent to each another
adj*n*	indicates a search for two terms where they appear within *n* words of each another

	Searches
1	diphosphonates/ or alendronate/ or ibandronic acid/ or risedronic acid/ or zoledronic acid/ or etidronic acid/ or pamidronate/
2	diphosphon$.ti,ab,kf.
3	bisphosphon$.ti,ab,kf.
4	alendron$.ti,ab,kf.
5	fosamax.ti,ab,kf.
6	risedron$.ti,ab,kf.
7	actonel.ti,ab,kf.
8	zoledron$.ti,ab,kf.
9	aclasta.ti,ab,kf.
10	ibandron$.ti,ab,kf.
11	etidron$.ti,ab,kf.
12	pamidron$.ti,ab,kf.
13	or/1-12
14	attitude/
15	attitude of health personnel/
16	exp attitude to health/ [includes patient satisfaction and patient preference]
17	choice behavior/
18	decision making/
19	attitud$.ti,ab,kf.
20	percept$.ti,ab,kf.
21	expectation$.ti,ab,kf.

22	experienc$.ti,ab,kf.
23	preferen$.ti,ab,kf.
24	choice$.ti,ab,kf.
25	belie$.ti,ab,kf.
26	opinion$.ti,ab,kf.
27	priorit$.ti,ab,kf.
28	benefi$.ti,ab,kf.
29	reason$.ti,ab,kf.
30	decision$.ti,ab,kf.
31	motiv$.ti,ab,kf.
32	justif$.ti,ab,kf.
33	(concern or concerns or concerned).ti,ab,kf.
34	(view or views or viewed).ti,ab,kf.
35	satisf$.ti,ab,kf.
36	value$1.ti,ab,kf.
37	or/14-36
38	Qualitative Research/ [After DeJean et al., 2016. Qual Health Res **26**(10): 1307-1317]
39	interview/
40	(theme$ or thematic).mp.
41	qualitative.af.
42	nursing methodology research/
43	questionnaire$.mp.
44	ethnological research.mp.
45	ethnograph$.mp.
46	ethnonursing.af.
47	phenomenol$.af.
48	(grounded adj (theor$ or study or studies or research or analys?s)).af.
49	(life stor$ or women$ stor$).mp.
50	(emic or etic or hermeneutic$ or heuristic$ or semiotic$).af.
51	((data adj1 saturat$) or participant observ$).tw.
52	(social construct$ or postmodern$ or post modern$ or poststructural$ or post structural$ or feminis$ or interpret$).mp.
53	(action research or cooperative inquir$ or co operative inquir$).mp.
54	(humanistic or existential or experiential or paradigm$).mp.

55	(field adj (study or studies or research)).tw.
56	human science.tw.
57	biographical method.tw.
58	theoretical sampl$.af.
59	((purpos$ adj4 sampl$) or (focus adj group$)).af.
60	(account or accounts or unstructured or open ended or text$ or narrative$).mp.
61	(life world or conversation analys?s or personal experience$ or theoretical saturation).mp.
62	((lived or life) adj experience$).mp.
63	cluster sampl$.mp.
64	observational method$.af.
65	content analysis.af.
66	(constant adj (comparative or comparison)).af.
67	((discourse$ or discurs$) adj3 analys?s).tw.
68	narrative analys?s.af.
69	heidegger$.tw.
70	colaizzi$.tw.
71	spiegelberg$.tw.
72	van manen$.tw.
73	van kaam$.tw.
74	merleau ponty.tw.
75	husserl$.tw.
76	foucault$.tw.
77	(corbin$ adj2 strauss$).tw.
78	glaser$.tw.
79	(mix$ adj2 (method$ or design$)).af. [filter amended to identify mixed method studies]
80	or/38-79
81	13 and 37 and 80

Supplementary material 3. Subtheme descriptions and illustrative key findings

Main theme	Subtheme	Description	Illustrative Key findings
Intervention Coherence	Necessity	Both patient and clinician participants described osteoporosis, falling and fracturing as a normal part of ageing and this view was associated with the perception that medication or treatment was futile.[16,30] One GP described the 'problem is not with the treatment, it's with the diagnosis': perceiving that the indications for treatment had broadened over recent years.[40] The absence of symptoms was reported by clinicians as a disincentive to patients accepting treatment,[36,43] however, patients questioned whether osteoporosis really was asymptomatic.[23] Patient participants who conceptualised osteoporosis as having consequences, e.g. as a cause of disability including 'shrinking' and 'stooping', were motivated to take medication.[29] Patient participants described other ways of controlling their condition and preventing fracture, for example, by not falling.[42]<br>In some patients who initiated treatment, the notion of osteoporosis as a chronic disease was noted not to make sense with the need to take bisphosphonate medication for 5 years.[23]	Patients perceived minimal susceptibility to the negative consequence of osteoporosis in the future and did not consider osteoporosis to be a serious health condition.[35]<br>Avoiding consequences (including shrinking, stooping, fractures) of osteoporosis was a strong motivator for adherence in PMW.[29]
	Concerns	Before starting bisphosphonates, patients noted concern and fear of bisphosphonate-specific side effects. This could be informed by vicarious experience of a family member,[41] or information from the media.[29] The special instructions for use, the limited duration of treatment and the name 'acid' were all cited as reasons underlying the perception that bisphosphonates must be harmful. Both patients and HCP's also cited a mistrust of pharmaceutical companies,[30,36,40] or a general aversion to drugs.[29,40,42,44]	{Women} were concerned about the long lists of drug side effects in advertisements.[16]<br>'Once you're on it, then it stays in your system and you wonder what damage have you've done to yourself?'[16]<br>Some PMW did not like the idea of taking any medications because they viewed medications as artificial and thought they had unpredictable effects.[29]
	Perceptions of own health	Some patients reported a perception that they were healthy, with some disbelieving they had osteoporosis and/or high fracture risk, and therefore and would reject medication and a label of a disease.[29] Conversely, others conceptualised bisphosphonates as a	Some patients initiated bisphosphonates to stay healthy.[33]<br>For PMW who considered themselves healthy, the idea of

For peer review only

		mechanism to remain healthy[33] and/or autonomous.[30] In a study of French GPs, on respondent also suggested patients wanted to know how to '"age well".[36]	medication was disconcerting as it meant perceiving themselves as sick.[29]
	Decision process	Across studies patients and HCPs described perceptions that the benefits did not outweigh the risks.[16,33,39,42] Often in these descriptions, the value of treatment was not clearly articulated meaning this assessment meant the patient weighing up staying as they were, or experiencing new side effects.[30] However, even when the risk of fracture was acknowledged, medication could still be seen as something to avoid.[42] The opposing view that the 'benefits were worth the costs' was evident in circumstances where benefits were described.[29] Others studies with patients reported that this decision was 'difficult' with one participant describing it as like 'Russian roulette'.[46] Balancing necessity against concerns was influenced by contingent factors such as trust in the clinician and could either be an easy or difficult and ongoing process. Patient participants talked about 'confidence' and 'trust' in their HCP, which could be associated with minimal contemplation to take treatment, or alternatively mistrust, or a failure to be 'convinced'.[16,26,27,29,32] Some patients reported clinicians as being persistent in their recommendation to take bisphosphonates;[32] however, conversely, patients also described by dissuaded by their doctor against treatment.[28] Often, patients described seeking information from other sources to make the final decision which often resulted in a decision against treatment.[46]<br>For those who initiated medication, an ongoing re-assessment of risk and benefit was noted,[23,33,46] particularly in studies that employed longitudinal methods.[30,31,33] Patients reported their decision making was influenced by experiencing a future fracture,[46] follow-up scans,[26] experienced side effects,[29,30] views of others and other experienced illnesses or life events.[31]	For some, the decision to take bisphosphonate involved minimal contemplation because they liked/trusted their health care provider.[46]<br>Patients who found the decision difficult sought alternative sources of information (professional and non) which often resulted in decision not to take OP medication.[46]
	Ethicality	Both orthopaedic and primary care clinicians reported a 'bias' against treating the elderly due to a belief 'nothing can be done for them'.[16] However, some patients also perceived that they were too old to benefit.[42] HCPs were seen to use the using ethical principle of non-maleficence to justify not recommending bisphosphonates. They questioned the negative side effects 'for a benefit that has not really been proven' and worried about being blamed for causing their patients ill-health.[28,40] Patients, in some circumstances, doubted the beneficence of the health care professionals e.g. perceiving	Clinicians {primary care and specialists} report bias against treating elderly patients because of a general tendency to believe that nothing can be done for them.[16]

For peer review only

		their physician as a 'pill pusher' or the motivation for prescribing medication being to receive money in return.[32]	
Affective Attitudes	Emotions	Patients described wide-ranging fears including fear of common and rare side effects and fear of new side effects emerging in the future. Patients described fear of bisphosphonates staying in their system,[16] with one patient participant describing bisphosphonates as akin to chemicals used to clean machines.[23] Patients also worried information was being withheld, or were fearful of the sheer amount of information to take in.[29] Both clinicians and patients described media reports as the source of fear, with patients also citing experiences of friends and family.[29] Fear of addiction was mentioned by patients in one study.[23] Patients and HCPs also expressed annoyance with the special instructions associated with oral bisphosphonate use, and annoyance with experienced oesophageal side effects.[32]<br>In two studies, patient participants reported that they experienced feelings of safety and reassurance when taking bisphosphonates,[27] linked to the anticipated benefits.[29]	"..when I read the side effects it was like a *horror film* really".[30]<br><br>medication provided a feeling of safety and reassurance.[27]
Burden	Special instructions	The method of administration of oral bisphosphonates caused concern to patients, both prior to initiating treatment,[40] and whilst on the treatment,[28] causing disruption to daily life. The need to remain upright after taking the medication and only being allowed to drink water was burdensome, and led to some disregarding the administration requirements.[29] Specific activities that needed to be actioned first thing in the morning also competed with taking oral bisphosphonates, with patients citing examples such as the need to have a coffee or run a family errand early every morning.[46] Primary care physicians reported that taking bisphosphonates was a 'hassle' for patients.[16] The frequency of the oral bisphosphonates, once a week, led to a number of reports of patients forgetting to take their medication.[16,23,29,30,44] Varying reports were identified about whether daily or weekly regimes were more or less burdensome.[16,29] Four studies reported patients' perceptions that the special instructions were not disruptive or burdensome.[27,29,31,44]	Some patients were able to rearrange their daily routines to accommodate {bisphosphonate} requirements, but others would intentionally disregard the administration requirements or forget to take the medication if it did not fit into their schedules.[29]
	Side effects	Experienced side effects were discussed in three of the studies interviewing clinicians,[34,38,39] eight with patients[23,25,27,29-31,33,46] and five with mixed participants.[16,40-42,44] Experienced side effects were reported as a common reason for lack of adherence, with gastrointestinal disturbances being described as "horrendous	Gastrointestinal disturbances from taking bisphosphonates were most notable and were described as

		diarrhoea" and "wrecking my stomach.[29,44] Patients reported stopping medications after experiencing side effects, did not always disclose side effects to HCPs and noted that the treatment 'was almost more disabling than the disease'.[28,31,45]	"horrendous diarrhoea" and "wrecking my stomach.[29]
	Costs	Financial costs were discussed in five studies, four of which were conducted in North America and one in Australia.[16,29,38,41,45] Patients did not report cost as a barrier to bisphosphonates specifically, however, medical insurance was perceived by clinicians as a barrier due to its complexity.[38,41,44] Indirect costs relating to travel and the need for increased dental checks were mentioned briefly but not described as a problem.[25,45]	Cost was not a limiting factor to adherence if patients had insurance coverage for medications. Even patients without insurance expressed a willingness to make sacrifices to pay for the medications because they thought the benefits were worth the cost.[29] Providers {secondary care} stated that due to cost not being covered by insurance companies, patients stop taking or alter dose/frequency.[44]
Opportunity costs	Co-morbid conditions	Physicians perceived bisphosphonate treatment was less important to patients who might have other more pressing health conditions [25,39] particularly in the absence of symptoms.[31,36] Patients also reported that other health conditions took priority over their prescribed bisphosphate leading them not to start or discontinue medication.28 Within the time-limited consultation, multiple competing priorities relating to other health conditions was reported by HCPs, resulting in a 'pecking order', and less time to discuss bisphosphonates.[25,42]	(Bisphosphonates) are lower down in the pecking order of things that we look at when we are supervising polypharmacy, when we are looking at chronic disease management".[25]
Perceived effectiveness	Mechanism of effectiveness	*Mechanism of effectiveness:* Patients expressed confusion about how bisphosphonates work and uncertainty about whether they strengthen, prevent worsening or slow the decline in bone density.[26,27,44] Patients talked about bone density scans as providing 'proof' of whether their medication was effective, however, there were differing reports of whether stabilisation in density was considered as treatment success.[32,42] The lack of systematic reduction in fracture or improvement in bone density was noted to result in ambivalence about efficacy and importance.[42] Patients described wanting more explanation about, and evidence of effectiveness (including quantified benefit).[16,23,29,30,32] Prior to initiating treatment, the perceived effectiveness of bisphosphonates was influenced in patients primarily by vicarious experience of friends	Taking anti-osteoporosis drugs was noted to not always seem to lead to improvement in their bone density and did not systematically prevent fracture.[42]

For peer review only

		or relatives.[32,40,41] Examples of relatives who had fractured on treatment or had hip or knee joint replacements were given as examples of lack of efficacy.[40]<br>Patients cited clinicians not meeting their informational needs about effectiveness, which may have been due to their own reported doubts.[39,40] Other clinicians expressed continued doubts about effectiveness in specific populations (e.g. the elderly) or in in relation to fracture risk at specific sites.[42] Patients in one study reported being told by health care professionals bisphosphonates are not effective for everyone[24] and in one study, clinicians questioned predictors of response.[39]	
	Monitoring and follow-up	Follow-up and monitoring were reported by clinicians[37] and patients[30] to support adherence to oral treatment, but generally felt to be lacking in primary care, in part due to uncertainties about who, when and what to monitor.[37] Patients reported not feeling supported with continued persistence with treatment[30] and reported the need for more reviews, feedback and help with 'ways to keep going' with medications.[16,23,30]	Women anticipated the next DXA scan as being the "proof" of whether the treatment was effective.[31] Reviewing patients' BMD results with them helped them evaluate the status of their osteoporosis, which motivated them to either start or continue taking their medicine.[29]
Self-efficacy	Supporting routinisation	*Supporting routinisation* Being able to successfully follow the special instructions for taking oral bisphosphonates, and incorporate the regime into daily routines appeared to be important to acceptability.[44] Other reported strategies to support self-efficacy were using pill compartments and calendar systems/reminders.[16] Patients reported that HCPs should supplement their oral instructions about BP administration with written ones.[44] Information, support and encouragement was needed throughout treatment but felt to be lacking by patients[16,30,46]. Patients and HCPs reported insufficient time in consultations to cover all the information about bisphosphonate medication.[42,44]	Patients noted that tips for routinizing medication use, such as using triggers (e.g., meals, calendars, placement of medications) to remember when to take medications, facilitated long-term adherence.[16]
	HCP knowledge and attitudes	Primary care providers did not feel confident in their own knowledge about bisphosphonates; they described guidelines as confusing and too detailed, expressing a number of uncertainties relating to who to start medication in, how long to continue medication for, the relationship between bisphosphonates and co-dependency for calcium/vitamin D, safety, when treatment should be changed including dose.[16,26,39,42] Some primary care clinicians indirectly suggested perceptions that osteoporosis was not a priority. Secondary care providers suggested osteoporosis champions in primary care would help educate primary care clinicians who were less interested in the condition.[25,44] It was also reported that non-medical clinicians	Physicians reported need for training in treating and help with therapeutic decision making.[42]

For peer review only

		(pharmacists or nurses) may be more knowledgeable or have more time to discuss bisphosphonates.[25,44]	
	Service level barriers	In terms of professional roles, clinicians in two studies described uncertainty about whose role it was to start and monitor treatment.[16,37] This was compounded by perceived poor communication between primary and secondary care, including update of the patients prescriptions on the electronic medical record.[44] Further reported barriers to treatment included lack of incentivisation[37] difficulty ordering, accessing or interpreting investigations to monitor treatment,[16,39] external restrictions on prescribing and access to intravenous bisphosphonates[37] and lack of time in primary care consultations.[16]	Provider barriers to treatment include lack of knowledge, other priorities, limited access and limited time.[43]<br>GPs regretted the absence of consensus about the professional in charge of osteoporosis.[28]<br>A number of participants {HCPs/managers} thought that intravenous zoledronic acid should be more widely available to improve adherence.[37]

For peer review only

Acceptability of bisphosphonates among patients, clinicians and managers: a systematic review and framework synthesis.

Journal:	*BMJ Open*
Manuscript ID	bmjopen-2020-040634.R1
Article Type:	Original research
Date Submitted by the Author:	01-Sep-2020
Complete List of Authors:	Paskins, Zoe; Keele University, School of Primary Community and Social Care; Haywood Hospital, Academic Rheumatology Centre<br>Crawford-Manning, Fay; Keele University, School of Primary Community and Social Care; Haywood Hospital, Academic Rheumatology Centre<br>Cottrell, Elizabeth ; Keele University, School of Primary, Community and Social Care<br>Corp, Nadia; Keele University, School of Primary, Community and Social Care<br>Wright, Jenny; Keele University, School of Primary Community and Social Care<br>Jinks, Clare; Keele University, School of Primary, Community and Social Care<br>Bishop, Simon; University of Nottingham, Centre for Health Innovation, Leadership and Learning<br>Doyle, Alison; Royal Osteoporosis Society<br>Ong, Terence; University of Malaya, Faculty of Medicine<br>GITTOES, NEIL; University of Birmingham, Centre for Endocrinology Diabetes and Metabolism<br>Leonardi-Bee, Jo; University of Nottingham, Faculty of Medicine & Health Sciences<br>Langley, Tessa; University of Nottingham, Division of Epidemiology and Public Health<br>Horne, Robert; University College London, School of Pharmacy<br>Sahota, Opinder; Nottingham University Hospitals NHS Trust, Department of Geriatric Medicine
**Primary Subject Heading**:	Rheumatology
Secondary Subject Heading:	Health services research, Patient-centred medicine
Keywords:	Rheumatology < INTERNAL MEDICINE, Bone diseases < ORTHOPAEDIC & TRAUMA SURGERY, Musculoskeletal disorders < ORTHOPAEDIC & TRAUMA SURGERY

For peer review only - http://bmjopen.bmj.com/site/about/guidelines.xhtml

I, the Submitting Author has the right to grant and does grant on behalf of all authors of the Work (as defined in the below author licence), an exclusive licence and/or a non-exclusive licence for contributions from authors who are: i) UK Crown employees; ii) where BMJ has agreed a CC-BY licence shall apply, and/or iii) in accordance with the terms applicable for US Federal Government officers or employees acting as part of their official duties; on a worldwide, perpetual, irrevocable, royalty-free basis to BMJ Publishing Group Ltd ("BMJ") its licensees and where the relevant Journal is co-owned by BMJ to the co-owners of the Journal, to publish the Work in this journal and any other BMJ products and to exploit all rights, as set out in our licence.

The Submitting Author accepts and understands that any supply made under these terms is made by BMJ to the Submitting Author unless you are acting as an employee on behalf of your employer or a postgraduate student of an affiliated institution which is paying any applicable article publishing charge ("APC") for Open Access articles. Where the Submitting Author wishes to make the Work available on an Open Access basis (and intends to pay the relevant APC), the terms of reuse of such Open Access shall be governed by a Creative Commons licence – details of these licences and which Creative Commons licence will apply to this Work are set out in our licence referred to above.

Other than as permitted in any relevant BMJ Author's Self Archiving Policies, I confirm this Work has not been accepted for publication elsewhere, is not being considered for publication elsewhere and does not duplicate material already published. I confirm all authors consent to publication of this Work and authorise the granting of this licence.

For peer review only

Title: **Acceptability of bisphosphonates among patients, clinicians and managers: a systematic review and framework synthesis.**

Corresponding Author: Dr. Zoe Paskins, School of Primary Community and Social Care, Keele University, David Weatherall Building, Newcastle-under-Lyme, UK, ST5 5BG and Haywood Academic Rheumatology Centre, Haywood Hospital, High Lane, Burslem, Stoke-on-Trent ST6 7AG.

z.paskins@keele.ac.uk, 01782 733975

Author List:

Dr. Zoe Paskins, School of Primary, Community and Social Care, Keele University & Haywood Academic Rheumatology Centre, Stoke-on-Trent, UK

Dr. Fay Crawford-Manning, School of Primary, Community and Social Care, Keele University & Haywood Academic Rheumatology Centre, Stoke-on-Trent, UK

Dr. Elizabeth Cottrell, School of Primary, Community and Social Care, Keele University, Newcastle-under-Lyme, UK

Dr. Nadia Corp, School of Primary, Community and Social Care, Keele University, Newcastle-under-Lyme, UK

Dr. Jenny Wright, School of Primary, Community and Social Care, Keele University, Newcastle-under-Lyme, UK

Prof. Clare Jinks, School of Primary, Community and Social Care, Keele University, Newcastle-under-Lyme, UK

Dr. Simon Bishop, Centre for Health Innovation, Leadership and Learning, University of Nottingham, Nottingham, UK

Alison Doyle, Royal Osteoporosis Society, Bath, UK

Dr. Terence Ong, Department of Medicine, Faculty of Medicine, University of Malaya, Kuala Lumpar, Malaysia

For peer review only

Prof. Neil Gittoes, Centre for Endocrinology Diabetes and Metabolism, University of Birmingham, Birmingham, UK

Prof. Jo Leonardi-Bee, Faculty of Medicine & Health Sciences, University of Nottingham, Nottingham, UK

Associate Prof. Tessa Langley, Division of Epidemiology and Public Health, University of Nottingham, Nottingham, UK

Prof. Robert Horne, School of Pharmacy, University College London, London, UK

Prof. Opinder Sahota, Department of Geriatric Medicine, Nottingham University Hospitals NHS Trust, Nottingham, UK

Key Words:

Acceptability; Theoretical Framework of Acceptability, Bisphosphonates; Osteoporosis

Current word count: 4097

For peer review only

Abstract (299/300 words):

OBJECTIVE: To explore the acceptability of different bisphosphonate regimens for the treatment of osteoporosis among patients, clinicians and managers, payers and academics.

DESIGN: A systematic review of primary qualitative studies. Seven databases were searched from inception to July 2019. Screening, data extraction and quality assessment of full-articles selected for inclusion were performed independently by two authors. A framework synthesis was applied to extracted data based on the Theoretical Framework of Acceptability (TFA). The TFA includes seven domains relating to sense-making, emotions, opportunity costs, burden, perceived effectiveness, ethicality and self-efficacy. Confidence in synthesis findings was assessed.

SETTING: Any developed country healthcare setting

PARTICIPANTS: Patients, healthcare professionals, managers, payers and academics

INTERVENTION: Experiences and views of oral and intravenous bisphosphonates.

RESULTS: Twenty-five studies were included, mostly describing perceptions of oral bisphosphonates. We identified, with high confidence, how patients and HCPs make sense (coherence) of bisphosphonates by balancing perceptions of need against concerns, how uncertainty prevails about bisphosphonate perceived effectiveness and a number of individual and service factors that have potential to increase self-efficacy in recommending and adhering to bisphosphonates. We identified, with moderate confidence, that bisphosphonate taking induces concern, but has the potential to engender reassurance, and that both side effects and special instructions for taking oral bisphosphonates can result in treatment burden. Finally, we identified with low confidence that multi-morbidity plays a role in people's perception of bisphosphonate acceptability.

CONCLUSION: By using the lens of acceptability, our findings demonstrate with high confidence that a theoretically informed, whole-system approach is necessary to both understand and improve adherence. Clinicians and patients need supporting to understand the need for bisphosphonates, and clinicians need to clarify to patients what constitutes bisphosphonate treatment success. Further research is

needed to explore perspectives of male patients and those with multi-morbidity receiving bisphosphonates, and patients receiving intravenous treatment.

Strengths and Limitations

- Comprehensive search strategy
- Robust framework synthesis underpinned by theory
- Inclusion of clinician and manager views in addition to patient perspectives.
- Use of GRADE-CERQual to give confidence in findings
- Qualitative studies reviewed for inclusion were frequently not specific about the anti-osteoporosis drugs participants were taking, meaning we may have missed papers or over-interpreted findings

For peer review only

INTRODUCTION/BACKGROUND

Osteoporosis is a disease that is characterised by skeletal fragility and changes in bone microarchitecture resulting in increased risk of fractures with no or low trauma.[1] The management and care of people with low trauma or fragility fractures results in considerable societal economic burden, annual cost in the UK alone is £4.4 billion.[2] Furthermore, the personal impact of fragility fractures is considerable, with potential deleterious effects on physical and psychological health, ability to live independently and increased risk of death. Many of these fractures are potentially preventable with appropriate cost- and clinically-effective drug treatments such as bisphosphonates, the mainstay of osteoporosis treatment. However, the success of treatment depends on patients initiating (starting), executing (or implementing - taking correctly) and persisting (continuing) medication; collectively these processes are described as adherence. Adherence with osteoporosis medications is notoriously poor and reported to be poorer than other disease areas. Oral bisphosphonate persistence rates at 1 year are commonly estimated between 16 and 60%.[3] Worldwide, many people who would benefit from osteoporosis drugs are not receiving them, and this treatment gap has been described as an 'osteoporosis crisis'.[4] The treatment gap is compounded by poor adherence which results in potentially preventable fragility fractures with their associated burden for patients and their carers, difficulties in professional-patient relationships, and wasted healthcare resources.[5]

There are a number of different bisphosphonates, some are administered orally others intravenously. A variety of regimes in terms of dose frequency also exists. Alendronic acid, an oral once-weekly bisphosphonate, is considered first-line and most commonly used.[6] Bisphosphonates work to reduce fracture risk. A recent network meta-analysis demonstrated that bisphosphonate treatment reduces the risk of fragility fracture (depending on site) by 33-54%.[7] Oesophageal or gastrointestinal related side effects are the most common adverse effects of oral bisphosphonate use. To counter these, patients taking oral bisphosphonates are required to remain upright and fast for half an hour after ingestion. Rare side effects of bisphosphonates include osteonecrosis of the jaw and atypical femur fractures, both of which have received significant media attention. Such media reports are temporally related to declining bisphosphonate use.[7] Due to the gastrointestinal

For peer review only

side effects and special instructions for taking oral treatment, it has been suggested that alternative bisphosphonate regimens, for example annual intravenous zoledronic acid, may promote long-term adherence.[8-11] Studies to date which have examined patient preferences for osteoporosis treatment, suggest that patients prefer injections given less frequently;[12-14] however, research in other chronic diseases shows that although adherence is improved with less frequent medications and that patients prefer oral to injection treatment.[15] In osteoporosis, the majority of studies that explore patient preferences employ quantitative methods, for example, discrete choice experiments where patients are asked to choose between hypothetical treatments in regards to various attributes (e.g. efficacy, side effects, route and frequency of administration).[13] Such studies cannot provide comprehensive insight into patient views, experiences or the explanations for these preferences.

In order to fully understand the osteoporosis treatment gap, and ultimately improve adherence, it is important to understand perspectives of all relevant stakeholders: patients, healthcare professionals (HCPs), managers, payors and academics.[16,17] This can be achieved using the lens of 'acceptability', defined as "a multi-faceted construct that reflects the extent to which people delivering, or, receiving a healthcare intervention consider it to be appropriate, based on anticipated or experienced cognitive and emotional responses to the intervention".[18,19] In the context of a research program designed to determine the research agenda for optimising bisphosphonate treatment, the primary aim of this systematic review is to explore the acceptability of different bisphosphonates regimens among patients, and clinicians and managers.

METHODS

We conducted a systematic review and framework synthesis of qualitative studies exploring patient and clinician views and experiences of bisphosphonates. The conduct and reporting of this review followed the Preferred Reporting Items for Systematic Reviews and Meta-Analyses (PRISMA) guidelines (see supplementary file 1 for PRISMA checklist). The protocol of the systematic review is registered in PROSPERO [CRD42019143526].

For peer review only

Eligibility

To be eligible for inclusion, studies needed to report on patients', clinicians', academics', and/or manager/payers' experiences and preferences regarding bisphosphonate regimes for adults (≥ 18 years) with osteoporosis. Bisphosphonates needed to be mentioned by name, or there needed to be sufficient information that was specific to bisphosphonate (e.g. reference to the special instructions for use of oral bisphosphonates), to deduce that study findings related to bisphosphonates, as agreed by two clinically experienced authors independently. Papers describing experiences of osteoporosis more generally were included if there were findings relating to bisphosphonate treatment in the study abstract. Studies were only included if they were qualitative in design, or mixed methods with a qualitative component, relevant to a developed country setting and written in English language. Studies were excluded that involved paediatric patients; patients and clinicians receiving/recommending other treatments for osteoporosis; and studies in which bisphosphonates were being used for other indications (e.g. malignancy or Paget's disease).

Search Methods

Systematic searches were conducted in 7 bibliographic databases (MEDLINE, EMBASE, AMED, CINAHLPlus, PsycINFO, ASSIA, and Web of Science [Social Science Citation Index (SSCI) and Conference Proceedings Citation Index- Social Science & Humanities (CPCI-SSH)] from inception to 15th July 2019. The search strategy utilised database subject headings and text word searching in title, abstract or keywords, combining terms for: 1) bisphosphonates; 2) experiences and preferences; and 3) qualitative research, based on DeJean et al.'s search filter (see supplementary file 2 for full MEDLINE search strategy).[19] Search terms were adapted as appropriate for each database platform.

In addition, grey literature was searched (DART Europe, Open Grey, and NDLTD (National Digital Library of Theses and Dissertations)); the reference lists of all included studies and relevant systematic reviews identified were checked and key studies were citation tracked.

Study Selection

For peer review only

Two-stage screening of articles against eligibility criteria was undertaken. Firstly, titles and abstracts were screened, then full texts. At both stages screening was conducted by sets of two reviewers independently (NC, EC, ZP) and articles were excluded by agreement. Disagreements were resolved through discussion or by third reviewer adjudication.

Data extraction

For each paper data extraction was completed independently by two researchers (ZP and JW or EC and FM). Key findings from the results sections of papers relating to bisphosphonates were extracted; a 'key finding' was defined as any sentence or statement relating to views or experiences of bisphosphonates from the results section of the paper or abstract. Wherever possible, the key finding was extracted as written by the author, with minimal edits only for clarification, description of context or for consistency across papers. For each paper, two authors extracted key findings independently, and subsequently agreed a final list of key findings for each paper. Data were also extracted on participant numbers and demographics, data collection technique, setting and country. Additionally, if available for patients, information was extracted on their bisphosphonate use including type of drug and current status (adherent, non-adherent, decliner).

Quality appraisal

The quality of each study was assessed using the CASP qualitative tool. This tool consists of 10 items split into 3 sections (qualitative suitability, data analysis and overall quality) (Supplementary File 2). The first two sections consist of items related to qualitative suitability and data analysis, which were evaluated as "yes", "no", "unclear" or "partial". The final question was an assessment based on the overall quality of the paper; this was informed by response to the previous items (indicating methodological quality) and by the relevance of the study to the review objectives and was rated as "high", "moderate" or "low". All papers were quality appraised by two researchers independently (FM, SB, JW). Disagreements were resolved through discussion with a fourth reviewer (ZP).

Synthesis

We used a framework synthesis approach informed by the 'Best Fit' model described by Carroll et al.[20] The "best fit" method offered a means to test, reinforce and build

For peer review only

on an existing published model, conceived for a different but relevant purpose. This approach was chosen as a published theory was identified from the literature that conceptualised acceptability - the Theoretical Framework of Acceptability (TFA).[18] The TFA is a relatively new framework which was developed to inform the understanding of acceptability of complex interventions, and consists of seven constructs: affective attitudes - the emotions elicited by an intervention; intervention coherence - the extent to which an intervention makes sense; perceived effectiveness - the perceived extent to which intervention will achieve purpose; burden – the amount of effort required to participate in an intervention; self-efficacy – individual's confidence that they can perform the behaviour(s) required to participate in the intervention; opportunity-costs - the extent to which benefits, profits, or values must be given up to engage in an intervention; and ethicality – the extent to which an intervention has a good fit with an individual's values. The framework also incorporates temporal perspectives on *anticipated* and *experienced* acceptability at three time points before (prospective), during (experienced) and after (retrospective) experience of an intervention.

The TFA has not previously been used to evaluate drug acceptability. We anticipated the seven constructs of the TFA would be relevant to engagement with drug treatment; for example, burden could relate to treatment burden associated with administrating the drug or side effects. However, one aspect which did not appear to be explicitly conceptualised within the framework was patient beliefs about medicines. Studies across a range of long term conditions, healthcare systems and cultures have consistently shown that engagement with treatment is influenced by patients' personal evaluation of the medicine in question.[21] Particularly important is how they judge their personal need for treatment relative to their concerns about it. For this reason, we therefore included the Necessity Concerns Framework (NCF) [21], to further explore the TFA domain relating to intervention coherence.

The first author initially conducted inductive open coding on the data extracted, before mapping the codes to a draft framework derived from a priori themes (the domains of the TFA). Authors then met to first discuss the themes and compare findings for each study and the 'fit' to the draft framework. A preliminary synthesis was achieved using tabulation of studies, organising the studies into groups relating

For peer review only

to temporal perspectives and research question, and exploring relationships between studies and between groups.

A final coding framework was agreed at a second meeting of authors. A second author (FM) recoded the original key findings, where necessary, to the new framework to ensure all findings were represented. Finally, relationships between themes and TFA and NCF domains were explored by further group discussion. We used the Grades of Recommendation, Assessment, Development, and Evaluation Confidence in the Evidence from Qualitative Reviews (GRADE-CERQual) approach to determine confidence in our synthesised findings.[22]

Patient and Public Involvement

Members of the Nottingham National, Royal Osteoporosis Society Support Group were involved in a series of meetings to discuss the design of the overarching research programme in which this study sits, and confirmed that understanding acceptability of bisphosphonates from a range of perspectives was important. Patient were not directly involved in the conduct of this study.

RESULTS

The literature search identified 2040 unique articles, of which 25 met eligibility criteria (Figure 1), a summary of the studies is shown in Table 1.

The included studies were categorised into three groups: perceptions of osteoporosis generally; [23-29] healthcare service delivery issues unrelated to osteoporosis (de-prescribing) [30] and inter-professional communication in primary care[31]) and studies specific to osteoporosis treatments. The latter group was further subdivided into: those examining treatment barriers;[16,32-36] adherence;[37-39] decision-making;[40-44] or bisphosphonate-related side effects.[45,46] Only one study examining adherence and one examining decision making had research questions which specifically related to bisphosphonates.[38,43]

The majority (23) of studies were conducted in North America or Europe. Eighteen studies explored patient views,[16,23-27,33,35,37-46] of which eight included males, and one study recruited patients taking anti-osteoporosis drugs for glucocorticoid-induced osteoporosis [36]. Twelve studies explored HCPs' views,[16, 28-32,34-36,39,42,43,] and two studies interviewed managers.[16,34] No studies included

academic or payor participants. Of the 18 studies that included patients, 10 studies described how many of the patients were on anti-osteoporotic medication, however, only two reported the specific type of medication. Only one study reporting patient experience of receiving intravenous bisphosphonate.[27]

For peer review only

Table 1: Summary of included studies

First author and year	Participants[i]	Participant No. (male:female)	Bisphosphonate use and adherence[ii]	Data collection methods	Qualitative approach or analysis method[iii]	Recruitment setting	Country
**Studies in Group 1: Views of Osteoporosis**							
Besser 2012[23]	Pts	14 (0:14)	AOD unspecified	Interview	Framework analysis	One hospital	UK
Jaglal 2003[29]	HCPs<br>Family Physicians	32 (12:20)	N/A	Focus group	Constant Comparison	Primary Care	Canada
Otmar 2012[328]	HCPs<br>GP (n = 14), Practice nurse (n = 2)	16 (11:5)	N/A	Focus group	Analytic comparison<br>Constant comparison	Primary care	Australia
Sale 2015[24]	Pts	28 (2:26)	19/28 pts on AOD<br>adherent (n=19)<br>declined (n= 4)	Interview	Phenomenological study	National osteoporosis patient group	Canada
Sale 2010[25]	Pts	24 (6:18)	9/24 pts on AOD,<br>risedronate (n=8),<br>etidronate (n=1)	Focus group	Descriptive qualitative study	Fracture Clinic	Canada
Weston 2011[26]	Pts	10 (0:10)	AOD unspecified	Interview	Interpretative phenomenological analysis	Primary Care	UK
Hansen 2017[27]	Pts	15 (0:15)	AOD unspecified<br>adherent (n=12)<br>declined/stopped AOD (n=3)	Interview	Phenomenological hermeneutic approach	Women attending DXA at 2 hospitals	Denmark
**Studies in Group 2: Views of Osteoporosis Treatment (treatment barriers)**							
Alami 2016[35]	Mixed	Pts: 37 (0:37)<br>HCPs: 18 (8:10)	23/47 pts on AOD,<br>adherent (n=19)<br>declined/stopped AOD (n=18)	Focus group	Grounded theory	Hospital/community over 5 regions	France
Drew 2016[34]	HCPs<br>Nurse (n = 14), GP (n= 2), Specialists (n =17), Orthopaedic Surgeon (n = 4) Managers (n = 5), DXA technician (n=1)	43 (not given)	N/A	Interview	Thematic approach	11 hospitals in one region	UK
Feldstein 2008[16]	Mixed	Pts: 10 (0:10)<br>HCPs: 57 (not given)	AOD unspecified	Interview and focus group	Content analysis	Primary and secondary care	USA
Guzman-Clarke 2007[36]	HCPs	23(13:10)	24/100 pts on AOD	Focus group	Thematic content analysis	Urban academic Medical Centre	USA
Merle 2019(a)[32]	HCPs (GP)	16 (11:5)	N/A	Interview	Descriptive thematic analysis	Primary Care	France
Merle 2019(b)[33]	Pts	98 (53:45)	AOD Unspecified	Focus group	Inductive thematic analysis	Recruited from 2 existing research studies and community (medical insurance company)	France
**Studies in Group 2: Views of Osteoporosis Treatment (adherence)**							

Iversen 2011[39]	Mixed	Pts: 32 (2:30)<br>HCPs: 12 (5:7)	AOD unspecified	Focus group	Open coding (thematic analysis)	Secondary care	USA
Lau 2008[37]	Pts	37 (0:37)	33/37 pts on AOD, alendronate (n=9), etidronate (n=5), risedronate (n=19)	Focus group	Mixed phenomenological design	Primary care, secondary care and community pharmacies	Canada
Salter 2014[38]	Pts	30 (0:30)	20/30 pts on AOD<br>adherent (n=19)<br>declined (n=1)<br>stopped AOD (n=10)	Interview	Framework analysis	Primary Care	UK
	**Studies in Group 2: Views of Osteoporosis Treatment (Decision Making)**						
Mazor 2016[40]	Pts	36 (0:36)	15/36 pts on AOD<br>adherent (n=15)<br>declined (n=10)<br>stopped (n=11)	Telephone Interview	(thematic analysis)	Primary Care	USA
Sale 2011[44]	Pts	24 (6:15)	14/21 pts on AOD	Telephone Interview	Phenomenological study	Hospital based fracture screening programme	Canada
Swart 2018[42]	Mixed	Pts: 26 (4:22)<br>HCPs: 13 (not given)	10/26 pts on AOD<br>adherent (n=10)<br>declined (n=16)	Interview	Thematic analysis with elements of grounded theory	Recruited from a fracture prevention study	Netherlands
Scoville 2011[43]	Mixed	Pt: 18 (0:18)<br>HCP: 19 (12:7)	N/A	Videographic	(deductive checklist and descriptive)	Primary care (osteoporosis choice trial)	USA
Wozniak 2017[41]	Pts	12 (3:9)	7/12 pts on AOD,<br>adherent (n=7)<br>stopped (n=5)	Interview	Grounded theory	Recruited from a fracture prevention trial nested in secondary care	Canada
	**Studies in Group 2: Views of Osteoporosis Treatment (Bisphosphonate side effects)**						
Sturrock 2019[46]	Pts	23 (4:19)	13/23 pts on AOD	Interview	Grounded theory	Three regions including from secondary care	UK
Sturrock 2017[45]	Pts	17 (7:10)	N/A	Interview	Grounded theory	Primary Care	UK
	**Studies in Group 3: Non-Specific Osteoporosis Issues**						
Ailabouni 2016[30]	HCPs	10 GPs	N/A	Interview	Constant comparison	Primary Care	New Zealand
Sippli 2017[31]	HCPs	28 (6:22)	N/A	Interview	Content analysis	Primary Care	Germany

[i] Pts – patients; HCPs – healthcare professionals
[ii] Where specified. AOD – anti-osteoporosis drug. N/A not applicable
[iii] Text in parentheses – qualitative approach not explicitly stated

The findings related to quality appraisal are summarised in Table 2. The most common limitations of the included studies were lack of description of author reflexivity, lack of depth of analysis, use of normative statements and relatively small samples or studies conducted in a single site which may limit transferability of the findings. Furthermore, although the characteristics of the sample were generally reasonably described, in order to address our research question, we required information about medication use of participants which was frequently not described.

Using the CASP tool, 12 (48%) studies were scored as high value and the remaining 13 (52%) studies as moderate value. For 5/13 (38%) studies scored as moderate in value, this was due to methodological issues, and, for 8/13 (62%) studies this was because the focus of the paper was less relevant to our research question.

For peer review only

Table 2. Quality Appraisal

First author and year	*CASP tool Question*[i]										*Comments*[ii]
	**1**	**2**	**3**	**4**	**5**	**6**	**7**	**8**	**9**	**10**	
Group1: Views of Osteoporosis											
Besser 2012[23]	✓	✓	✓	p	✓		✓	p	✓	Moderate	Small sample, no mention of data saturation, limited to 'psychological' factors affecting adherence (discounting other factors by omission) and some use of normative statements
Jaglal 2003[29]	✓	✓	✓	✓	✓	u	✓	✓	✓	Moderate	Few findings relevant to our research question
Otmar 2012[28]	✓	✓	✓	✓	✓		✓	✓	✓	Moderate	Well conducted study, but limited findings relating to bisphosphonates
Sale 2015[24]	✓	✓	✓	✓	✓	u	✓	✓	✓	High	
Sale 2010[25]	✓	✓	✓	p	✓	u	✓	p	✓	Moderate	Small single site study, although data saturation reached. Language does not always appear to match approach (e.g. reporting patient 'inability' to link fractures to osteoporosis suggests prior normative assumptions)
Weston 2011[26]	✓	✓	✓	✓	✓	✓	✓	✓	✓	High	
Group 2: Views of Osteoporosis Treatment											
Alami 2016[35]	✓	✓	✓	✓	✓		✓	✓	✓	High	
Drew 2016[34]	✓	✓	✓	✓	✓	u	✓	✓	✓	High	
Feldstein 2008[16]	✓	✓	✓	✓	✓	u	✓	✓	✓	High	
Guzman-Clarke 2007[36]	✓	✓	✓	✓	✓	u	✓	u	✓	Moderate	Only partially relevant for our review given the focus on a specific population (Glucocorticoid-Induced Osteoporosis)
Merle 2019[32]	✓	✓	✓	p	✓	u	✓	u	✓	Moderate	Small sample (although data saturation reached) without attempt to structure to population and analysis lacks depth to answer our objective relating to bisphosphonate acceptability
Merle 2019[33]	✓	✓	✓	✓	✓	u	✓	✓	✓	Moderate	Limited information relevant to our research question in view of general focus on osteoporosis
Iversen 2011[39]	✓	✓	✓	p	✓		✓	p	✓	Moderate	Single centre study, although data saturation reached, limited information on coding/analysis and no discussion of findings with relevance to wider literature
Lau 2008[37]	✓	✓	✓	✓	✓		✓	✓	✓	High	
Salter 2014[38]	✓	✓	✓	✓	✓		✓	✓	✓	High	

For peer review only

Hansen 2017[27]	✓	✓	✓	✓	✓	✓	✓	✓	✓	High	
Mazor 2016[40]	✓	✓	✓	✓	✓	u	✓	u	✓	Moderate	Good relevance, single site. Descriptive approach without critical reflexivity or discussion of prior assumptions
Sale 2011[4446]	✓	✓	✓	✓	✓	u	✓	✓	✓	High	
Swart 2018[42]	✓	✓	✓	✓	✓	✓	✓	✓	✓	High	
Scoville 2011[43]	✓	✓	✓	✓	✓	u	✓	✓	✓	Moderate	Well conducted videographic study, but data coded against deductive categories of reasons to reject treatment, so limited potential to inform our objective about acceptability
Wozniak 2017[41]	✓	✓	✓	✓	✓	u	✓	✓	✓	High	
Sturrock 2019[46]	✓	✓	✓	✓	✓	u	✓	✓	✓	High	
Sturrock 2017[45]	✓	✓	✓	✓	✓		✓	✓	✓	Moderate	Aim only partially relevant to study question
Group 3: Non-specific Osteoporosis Issues											
Ailabouni 2016[30]	✓	✓	✓	p	✓	✓	✓	✓	✓	Moderate	Relatively small (10 respondents) study, although data saturation reached. Only partial relevant for current review with brief coverage of GPs views on discontinuing bisphosphonates in light of multimorbidities
Sippli 2017[31]	✓	✓	✓	✓	✓		✓	✓	✓	Moderate	Limited findings related to our research question

[i]CASP Quality assessment questions; 1, Was there a clear statement of the aims of the research? 2, Is a qualitative methodology appropriate? 3, Was the research design appropriate to address the aims of the research? 4, Was the recruitment strategy appropriate to the aims of the research? 5, Was the data collected in a way that addressed the research issue? 6, Has the relationship between researcher and participants been adequately considered? 7, Have ethical issues been taken into consideration? 8, Was the data analysis sufficiently rigorous? 9, Is there a clear statement of findings? 10, Value of study and relevance to review objectives. ✓ = Yes, u = Unsure, p = partial, blank = No.

[ii] Comments only made for those ranked Moderate or Low.

For peer review only

Fifteen individual sub-themes were identified which mapped to the seven domains of the TFA. Key findings relating to ethicality related to conflict between bisphosphonates and participants' values and were usually discussed as part of sense making. For this reason, issues relating to 'ethicality' were considered as part of 'intervention coherence', leaving six main themes, as shown schematically in Figure 2. Although it was possible to distinguish between two temporal perspectives, related to anticipated and experienced acceptability within most domains (with the exception of self-efficacy) the majority of anticipated acceptability findings related to intervention coherence.

The findings of the review are discussed below with GRADE-CERQual ratings of confidence in Table 3 and illustrative key findings for each theme/subtheme shown in Supplementary file 2. Subthemes are identified in the text in italics.

For peer review only

Table 3: GRADE-CERQual Summary of qualitative findings table

Review Finding [and contributing studies]	Methodological limitations	Coherence	Adequacy	Relevance	*CERQual Confidence assessment*
	Concerns				
**Intervention coherence:**<br>Both before starting, and during treatment, patients considered the perceived need or necessity for bisphosphonates based on their views of osteoporosis, including its seriousness and controllability, symptoms and their perception of their own health. Perceived need was weighed up against concerns about medication, including suspicion of drugs in general and specific concerns about bisphosphonate safety by both patients and HCPs. HCPs sometimes used principles of ethicality to support perceptions of low necessity and their reluctance to prescribe. The decision process of balancing necessity against concerns, was influenced by the doctor-patient relationship and wider societal influences including friends, family and the general media. This process influenced whether HCPs reported recommending bisphosphonates. For patients, the decision process could be explicit or tacit, was revisited over time and influenced both whether they initiated treatment and subsequently adhered.<br>[16,23,25-30,32,33,35-44,46]	*Minor*<br><br>12/22 papers rated moderate value due to sample size, depth of analysis or lack of reflexivity[i]	*None or very minor*<br><br>The finding reflects the complexity and variation of the data, and these influences on sense making are well supported by details in the underlying studies	*None or very minor*<br>22 papers contributed to this finding, and although some gave little detail, in-depth insights were reported in 10 papers and information was consistent across studies	*Minor*<br><br>Spread of studies from primary and secondary care and range of countries. Uncertainties remain about sense making related patients taking intravenous bisphosphonates and influence of gender	HIGH
**Perceived effectiveness**<br>Both patients and HCPs expressed doubt or uncertainty about the mechanism of effectiveness of bisphosphonates and expressed a range of treatment expectations including strengthening bone - improving bone density, preventing worsening of osteoporosis - maintaining bone density and/or total fracture prevention. Patients wanted proof or evidence of effectiveness through more structured monitoring and follow-up, and were disincentivised to continue treatment in the absence of evidence of perceived effectiveness.<br>[16,23,24,29,34,35,38-40,42,43]	*Minor*<br><br>7/15 papers rated moderate value, mostly (4/7) due to limited relevant content. Methodological concerns relate to depth of analysis or lack of reflexivity[i]	*None or very minor*<br><br>The finding reflects the complexity and variation of the data, and these issues are supported by details in the underlying studies	*None or very minor*<br>15 papers contributed to this finding. Some gave little detail, but in-depth insights were reported in 6 papers and information was consistent	*Minor*<br><br>Spread of studies from primary and secondary care and range of countries. Uncertainties remain about perceived effectiveness of intravenous bisphosphonates	HIGH
**Self-efficacy**<br>Measures to help patients integrate medication taking into daily routines (supporting routinisation), and the provision of information and support, enhanced their feeling of having control over their health and confidence to adhere to bisphosphonates. Clinician reported barriers to supporting adherence related to perceptions of their knowledge and attitudes, with several knowledge gaps and uncertainties reported, and the perception that osteoporosis was not a priority. Finally, service level barriers which impaired clinicians' self-efficacy in recommending and managing patients on bisphosphonates, included uncertainty	*Minor*<br><br>7/15 papers rated moderate value, mostly (4/7) due to limited relevant content. Methodological concerns relate to	*None or very minor*<br><br>The finding reflects the complexity and variation of the data, and these issues are supported by details in the underlying studies	*None or very minor*<br>17 papers contributed to this finding. Some gave little detail, but in-depth insights were reported in 5 papers and	*Minor*<br><br>Spread of studies from primary and secondary care and range of countries. Uncertainties remain about self-efficacy relating to	HIGH

For peer review only - http://bmjopen.bmj.com/site/about/guidelines.xhtml

about professional roles and responsibilities, capacity, access to intravenous drugs and communication and IT systems. [16,24,26,27,30-32,37,38,45]	depth of analysis or sample size[i]		information was consistent.	intravenous bisphosphonates	
**Affective attitudes:**<br>The emotions elicited by bisphosphonates were closely related to intervention coherence. Bisphosphonates were associated predominantly with negative emotions of fear (of side effects) and annoyance (with special instructions); however positive emotions of reassurance and hope were noted in two studies, linked to the anticipated protection that bisphosphonates could incur. [16,23,26,27,35,37,38,40]	*Minor*<br><br>2/8 papers rated moderate value due to depth of analysis or lack of reflexivity[i]	*None or very minor*<br><br>The finding reflects the data, supported by details in the underlying studies	*Moderate*<br><br>Reports of affective attitude were mostly descriptive with little depth	*Moderate*<br><br>Uncertainties remain about affective attitudes to injectable bisphosphonates received in hospital	MODERATE
**Burden:**<br>The burden or effort of oral bisphosphonates was described mostly relating to the special instructions to take oral bisphosphonates or experienced side effects, although costs incurred were also a potential source of burden.<br>[16,23,26,27,32,37-39,42,43,46]	*Minor*<br>4/11 papers rated moderate value due to sample size, depth of analysis[i]	*None or very minor*<br>The finding reflects the data, and these aspects of burden are supported by details in the underlying studies	*Moderate*<br>Reports mostly descriptive with little depth and a possible focus on presence of burden (side effects) rather than absence	*Moderate*<br>Uncertainties remain about burden of indirect costs (travel, dental checks) & burden due to intravenous bisphosphonates	MODERATE
**Opportunity costs**<br>Circumstances where competing priorities challenged adherence or initiation of bisphosphonates were described relating to co-morbid conditions. The presence of comorbid conditions were described as resulting in less time to support discussion about bisphosphonates in consultations and, result in recommendation of, and adherence to, bisphosphonates being given relative low priority. [16, 27,29,32,33,38, 41,42,44-46]	*None or very minor*<br>4/11 papers rated moderate value, but this was mostly (n=3) due to limited relevant content rather than methodological concerns.	*Moderate*<br>No discussion of the alternative explanation that having co-morbid conditions may facilitate bisphosphonate acceptability	*Moderate*<br>Reports were limited, lacked depth & 3 papers contained little content relevant to the research question	*Moderate*<br>No information about values, benefits that have to be given up to partake in intravenous bisphosphonates, which are likely to be different & likely limited sampling of patients with complex health needs	LOW

[i] Concerns considered minor because of the methodological strength of the other papers in this domain, & low likelihood that reflexivity would affect finding

For peer review only

Intervention Coherence (high confidence)

Both before starting, and during treatment, patients considered the perceived need or *necessity* for bisphosphonates based on their views of osteoporosis, including its seriousness and controllability, symptoms and their *perception of their own health.* Perceived need was weighed up against *concerns* about medication, including suspicion of drugs in general and specific concerns about bisphosphonate safety, by both patients and HCPs. HCPs sometimes used principles of *ethicality* to support perceptions of low necessity and their reluctance to prescribe. The *decision process* of balancing necessity against concerns, was influenced by the doctor-patient relationship and wider societal influences including friends, family and the general media. This process influenced whether HCPs reported recommending bisphosphonates. For patients, the decision process could be explicit or tacit, was revisited over time and influenced both whether they initiated treatment and subsequently adhered.

Perceived Effectiveness (high confidence)

Both patients and HCPs expressed doubt or uncertainty about the *mechanism of effectiveness* of bisphosphonates and expressed a range of treatment expectations including strengthening bone - improving bone density, preventing worsening of osteoporosis - maintaining bone density and/or total fracture prevention. Patients wanted proof or evidence of effectiveness through more structured *monitoring and follow-up,* and were disincentivised to continue treatment in the absence of evidence of perceived effectiveness.

Self-efficacy (high confidence)

Measures to help patients integrate medication taking into daily routines (*supporting routinisation*), and the provision of information and support, enhanced their feeling of having control over their health and confidence to adhere to bisphosphonates. Clinician reported barriers to supporting adherence related to perceptions of their *knowledge and attitudes,* with several knowledge gaps and uncertainties reported, and the perception that osteoporosis was not a priority. Finally, s*ervice level barriers* which impaired clinicians' self-efficacy in recommending and managing patients on

bisphosphonates, included uncertainty about professional roles and responsibilities, capacity, access to intravenous drugs and communication and IT systems.

Affective attitudes (moderate confidence)

The *emotions* elicited by bisphosphonates were closely related to intervention coherence. Bisphosphonates were associated predominantly with negative emotions of fear (of side effects) and annoyance (with special instructions); however positive emotions of reassurance and hope were noted in two studies, linked to the anticipated protection that bisphosphonates could incur.

Burden (moderate confidence)

The burden or effort of oral bisphosphonates was described mostly relating to the *special instructions* to take oral bisphosphonates or experienced *side effects*, although *costs* incurred were also a potential source of burden. Only one study included the experience of a patient on an intravenous bisphosphonate, this patient described low treatment burden as she only had to go once a year, and felt no side effects.[31]

Opportunity costs (low confidence)

There were few descriptions of 'benefits, profits, or values' being given up to take bisphosphonates. However, circumstances where competing priorities challenged adherence or initiation of bisphosphonates were described relating to *co-morbid conditions*. The presence of comorbid conditions was described as resulting in less time to support discussion about bisphosphonates in consultations and, result in recommendation of, and adherence to, bisphosphonates being given relative low priority.

For peer review only

DISCUSSION

This systematic review has used the lens of acceptability to understand perceptions of bisphosphonates and the problem of poor adherence. We have identified, with high confidence, how patients and HCPs make sense (coherence) of bisphosphonates by balancing perceptions of need against concerns, how uncertainty prevails about perceived effectiveness of bisphosphonates and how a number of individual and service factors have potential to increase self-efficacy in recommending and adhering to bisphosphonates. We identified with moderate confidence, that bisphosphonate taking induces fear, but has the potential to engender reassurance, and that both the side effects and special instructions for taking oral bisphosphonates can be a source of treatment burden. Finally, we identified with low confidence that multi-morbidity plays a role in people's perception of bisphosphonate acceptability.

To our knowledge, this is the first use of the Theoretical Framework for Acceptability, originally developed to evaluate acceptability of complex interventions, to evaluate the acceptability of medication. We explored the utility of the TFA from two perspectives, as an explanatory model for both patient and clinician acceptability and engagement. The TFA was useful for understanding and combining patient and clinician viewpoints; however, there was considerable overlap between domains; perceived efficacy, affective attitudes and self-efficacy beliefs are all likely to impinge on sense-making, or intervention coherence. The TFA alone does not provide a comprehensive framework for understanding patient acceptability or engagement with medicines, and of course it was not intended to do so. The sense-making aspect of the framework appeared pivotal, and the explanatory value of the framework was enhanced by the incorporation of the NCF to operationalise key engagement related beliefs. In the context of bisphosphonates, concern and associated fears predominate among patients, and perceived need may be underestimated if the consequences of osteoporosis and fragility fractures are not explained. In our findings, sense making was dynamic. Patients re-evaluated perceptions of bisphosphonates over time, expressing uncertainty relating to what represents successful treatment and citing perceived lack of effectiveness being cited as reason to discontinue. This is likely to be a particular problem for bisphosphonates, as opposed to other drugs commonly taken for prevention such as

For peer review only

statins and anti-hypertensive, where measures of feedback and effectiveness are more readily available.

The NICE guidelines for medicines adherence emphasises the need to take into account perceptions (e.g. necessity beliefs and concerns) and practicalities (e.g. capability and resources) that will affect individuals' motivation and ability to start and continue with treatment.[47] However, interventions designed to improve bisphosphonate adherence are often designed to 'educate' or persuade the patient of importance and are often not targeted to eliciting or addressing health beliefs, or informed by underpinning mechanisms of change.[3] There is therefore a need to ensure that any further design of interventions - to promote bisphosphonate adherence - draws on more comprehensive theoretical models of patient engagement with health conditions and medicines such as the Extended Common Sense Model.[48] This model situates individual's perceptions about drugs, and practical issues related to capability, in the context of illness and treatment representations.

Specifically, our findings suggest a need for clinicians to support patients to understand the need for treatment, to allay concerns where possible and to define what constitutes successful bisphosphonate treatment. Furthermore, clinicians need to support patients evaluate the advantages and disadvantages over time, given the dynamic nature of these decision processes.[48]

It is clear from our findings that clinicians also have necessity-concern dilemmas relating to bisphosphonates. A number of studies reported clinicians themselves perceiving low patient need, high concerns and perceptions treatment was not practical. This is perhaps in contrast with a previous quantitative study in asthma which demonstrated that clinicians held stronger positive beliefs about medicines than patients.[49] It is unclear to what extent the perceptions in our findings were generalisations or applied in specific circumstances, or to what extent these views were negotiated on an individual basis in discussion with patients. Problems may arise in the consultation if clinicians assume patients share their views and then may be less likely to explore patient perceptions of need or concerns. Furthermore, the limitations of interviewing HCPs are well documented; the accounts presented in an interview may not represent clinician underlying beliefs or behaviours meaning that

For peer review only

observational methods may be more appropriate to fully understand clinical decisional making.[50] Given the clinician has a pivotal role in sense making, interventions are also likely needed to address clinician knowledge, attitudes and beliefs. By including the views of clinicians and managers we have also identified a range of service level barriers to promoting bisphosphonate adherence relating to lack of clarity about professional roles, both across primary and secondary care, and within primary care, use of IT systems and access to intravenous treatments.

A strength of this review is the comprehensive search, use of underpinning theoretical framework, the inclusion of clinician views in addition to patients, and the use of the GRADE-CERQual to give confidence in our findings which has facilitated a clear identification of where further research is needed. Areas where we have identified moderate or low confidence are in need of further research and specifically relate to the influence of multi-morbidity on sense making, burden and self-efficacy in bisphosphonate users, the extent to which intravenous bisphosphonates may overcome issues related to treatment burden and self-efficacy, and the impact of bisphosphonates on affective attitudes and emotions. Furthermore, we have identified gaps in our understanding of how clinicians make decisions in practice, and how views of bisphosphonates may be influenced by gender. Given that many osteoporosis drugs have a different evidence base and licensing arrangements in men this is an area in need of further study.

The main limitation of this study relates to the lack of clarity in many of the included studies in the results sections about which osteoporosis treatments or bisphosphonates were being referred to, meaning that in some cases we may have over-interpreted findings relating to bisphosphonates that were about other osteoporosis drugs. However, all of our review findings were identified from comparison of data from several studies, and as bisphosphonates represent the mainstay of osteoporosis treatment, we consider that over-interpretation is unlikely. As there was frequently little detail about medication participants were taking or referring to, it is also possible that we have missed relevant studies. The views of males were underrepresented; although 8/18 studies included men, men represented less than 20% of the total patient population in the included studies. It is important for future studies to include males and specific populations such as those with glucocorticoid-induced osteoporosis who are likely to have different experiences

For peer review only

and needs.[51] Only two studies reported the views of managers but unfortunately neither of these studies distinguished professional roles in the presentation of results, so a further need exists to explore perceptions of this group, and perceptions of payors and academics. Finally, although the population from which each study sampled was reasonably well described, it was not always possible to appreciate if the setting was primary or secondary care; the majority of studies appeared to recruit from primary care which may explain the lack of findings related to intravenous bisphosphonates and limit the transferability of our findings to non-primary care settings.

CONCLUSIONS

In summary, using the lens of acceptability, we have identified the factors that influence how patients and clinicians make sense of bisphosphonates, described the experience of bisphosphonate taking in terms of burden and factors that both facilitate and hinder confidence in taking, and prescribing and monitoring bisphosphonates. Our findings demonstrate the need for a theoretically informed, whole-system approach' to enable clinicians and patients to get the best from bisphosphonate treatment. Patients need comprehensive support that takes account of the perceptions (e.g. treatment necessity beliefs and concerns) and practicalities (e.g. capability and resources) that influence their motivation and ability to start and continue with treatment. Clinicians need to moderate patient expectations and clarify what constitutes bisphosphonate treatment success. Finally, further research is needed to explore perspectives of managers, patients receiving intravenous bisphosphonates, men receiving bisphosphonates and the use of bisphosphonates in the context of multi-morbidity.

For peer review only

A. CONTRIBUTION STATEMENT

Conceptualisation: ZP, SB, EC, AD, TO, NG, JLB, TL, OS. Protocol: ZP, EC, NC, SB, LLB, TL, TO, OS, NG, AD. Search Implementation: ZP, EC, NC, JW, FCM. Data extraction and Quality: ZP, EC, NC, JW, FCM, SB. Synthesis: ZB, SB, EC, FCM, RH, CJ. Writing- Original draft: ZP, FCM. Writing- Review and Editing: ZP, SB, AD, TO, NG, JLB, CJ, TL, OS, FCM, JW, NC, NH, EC

B. CONFLICTS OF INTEREST

There are no competing interests for any author

C. FUNDING

This study is funded by the National Institute for Health Research (NIHR), [HTA NIHR127550]. ZP is funded by the NIHR, Clinician Scientist Award (CS-2018-18-ST2-010)/NIHR Academy. CJ is part funded by the NIHR Applied Research Collaboration West Midlands. The views expressed are those of the author(s) and not necessarily those of the National Health Service, the NIHR, or the Department of Health & Social Care.

D. DATA SHARING STATEMENT

All data relevant to the study are included in the article or uploaded as supplementary information

E. ACKNOWLEDGEMENTS

None to declare.

For peer review only

REFERENCES

1 Cooper C, Campion G, Melton LJ. Hip fractures in the elderly: A world-wide projection. *Osteoporos Int* 1992;2:285-9. doi:10.1007/BF01623184

2 National Osteoporosis Society. NHS RightCare scenario: The variation between sub-optimal and optimal pathways. *Rep* 2017:1–16.

3 Hiligsmann, M., Cornelissen, D., Vrijens, B. et al. Determinants, consequences and potential solutions to poor adherence to anti-osteoporosis treatment: results of an expert group meeting organized by the European Society for Clinical and Economic Aspects of Osteoporosis, Osteoarthritis and Musculoskeletal Diseases (ESCEO) and the International Osteoporosis Foundation (IOF). *Osteoporos Int* 2019;30:2155–65. https://doi.org/10.1007/s00198-019-05104-5

4 Khosla S, Shane E. A Crisis in the Treatment of Osteoporosis. *J Bone Miner Res* 2016;31:1485-7. doi:10.1002/jbmr.2888

5 May CR, Montori VM, Mair FS. We need minimally disruptive medicine. *BMJ* 2009;339:2830. doi:10.1136/bmj.b2803

6 National Institute for Health Care Excellence. Bisphosphonates for treating osteoporosis, Technology appraisal guidance (TA46) *NICE Guidance* 2017

7 Jha S, Wang Z, Laucis N, et al. Trends in Media Reports, Oral Bisphosphonate Prescriptions, and Hip Fractures 1996-2012: An Ecological Analysis. *J Bone Miner Res* 2015;30:2179-87 doi:10.1002/jbmr.2565

8 Recknor C, Czerwinski E, Bone HG, et al. Denosumab compared with ibandronate in postmenopausal women previously treated with bisphosphonate therapy: A Randomized Open-Label Trial. *Obstet Gynecol* 2013;121:1291-9. doi:10.1097/AOG.0b013e318291718c

9 Akarirmak Ü, Koçyiğit H, Eskiyurt N, et al. Influence of patient training on persistence, compliance, and tolerability of different dosing frequency regimens of bisphosphonate therapy: An observational study in Turkish patients with postmenopausal osteoporosis. *Acta Orthop Traumatol Turc 2016;50;415-23.* doi:10.1590/S0080-62342016000500008

For peer review only

10 Kishimoto H, Maehara M. Compliance and persistence with daily, weekly, and monthly bisphosphonates for osteoporosis in Japan: analysis of data from the CISA. *Arch Osteoporos* 2015;10:27. doi:10.1007/s11657-015-0231-6

11 Durden E, Pinto L, Lopez-Gonzalez L, et al. Two-year persistence and compliance with osteoporosis therapies among postmenopausal women in a commercially insured population in the United States. *Arch Osteoporos* 2017;12:22. doi:10.1007/s11657-017-0316-5

12 Saini SD, Schoenfeld P, Kaulback K, et al. Effect of medication dosing frequency on adherence in chronic diseases. *Am J Manag Care* 2009;15:22-33.

13 Hiligsmann M, Bours SPG, Boonen A. A Review of Patient Preferences for Osteoporosis Drug Treatment. *Curr Rheumatol Rep* 2015;17:61. doi:10.1007/s11926-015-0533-0

14 de Bekker-Grob EW, Essink-Bot ML, Meerding WJ, et al. Patients' preferences for osteoporosis drug treatment: A discrete choice experiment. *Osteoporos Int* 2008;19:1029-37. doi:10.1007/s00198-007-0535-5

15 Alten R, Krüger K, Rellecke J, et al. Examining patient preferences in the treatment of rheumatoid arthritis using a discrete-choice approach. *Patient Prefer Adherence* 2016;10:2217. doi:10.2147/PPA.S117774

16 Feldstein AC, Schneider J, Smith DH, et al. Harnessing stakeholder perspectives to improve the care of osteoporosis after a fracture. *Osteoporos Int* 2008;19:1527-40. doi:10.1007/s00198-008-0605-3

17 Bliuc D, Eisman JA, Center JR. A randomized study of two different information-based interventions on the management of osteoporosis in minimal and moderate trauma fractures. *Osteoporos Int* 2006;19:1309-17. doi:10.1007/s00198-006-0078-1

18 Sekhon M, Cartwright M, Francis JJ. Acceptability of healthcare interventions: An overview of reviews and development of a theoretical framework. *BMC Health Serv Res* 2017;17:88. doi:10.1186/s12913-017-2031-8

19 Dejean D, Giacomini M, Simeonov D, et al. Finding Qualitative Research

For peer review only

Evidence for Health Technology Assessment. *Qual Health Res* 2016;26:1307-17. doi:10.1177/1049732316644429

20 Carroll C, Booth A, Cooper K. A worked example of 'best fit' framework synthesis: A systematic review of views concerning the taking of some potential chemopreventive agents. *BMC Med Res Methodol* 2011:11:29. doi:10.1186/1471-2288-11-29

21 Horne R, Chapman SCE, Parham R, et al. Understanding patients' adherence-related Beliefs about Medicines prescribed for long-term conditions: A meta-analytic review of the Necessity-Concerns Framework. *PLoS One* 2013;8:e80633. doi:10.1371/journal.pone.0080633

22 Lewin S, Booth A, Glenton C, et al. Applying GRADE-CERQual to qualitative evidence synthesis findings: Introduction to the series. *Implement Sci* 2018;2. doi:10.1186/s13012-017-0688-3

23 Besser SJ, Anderson JE, Weinman J. How do osteoporosis patients perceive their illness and treatment? Implications for clinical practice. *Arch Osteoporos* 2012;7:115-24 doi:10.1007/s11657-012-0089-9

24 Sale JEM, Hawker G, Cameron C, et al. Perceived messages about bone health after a fracture are not consistent across healthcare providers. *Rheumatol Int* 2015;35:91-130. doi:10.1007/s00296-014-3079-y

25 Sale JEM, Beaton D, Sujic R, et al. 'If it was osteoporosis, i would have really hurt myself.' Ambiguity about osteoporosis and osteoporosis care despite a screening programme to educate fragility fracture patients. *J Eval Clin Pract* 2010;16:590-6. doi:10.1111/j.1365-2753.2009.01176.x

26 Weston JM, Norris E V., Clark EM. The invisible disease: Making sense of an osteoporosis diagnosis in older age. *Qual Health Res* 2011;21:192-704. doi:10.1177/1049732311416825

27 Hansen CA, Abrahamsen B, Konradsen H, et al. Women's lived experiences of learning to live with osteoporosis: A longitudinal qualitative study. *BMC Womens Health* 2017;17:17. doi:10.1186/s12905-017-0377-z

28 Otmar R, Reventlow SD, Nicholson GC, et al. General medical practitioners'

For peer review only

knowledge and beliefs about osteoporosis and its investigation and management. *Arch Osteoporos* 2012;7:104-14. doi:10.1007/s11657-012-0088-x

29 Jaglal SB, Carroll J, Hawker G, et al. How are family physicians managing osteoporosis?: Qualitative study of their experiences and educational needs. *Can Fam Physician* 2003;49:462-8.

30 Ailabouni NJ, Nishtala PS, Mangin D, et al. General practitioners' insight into deprescribing for the multimorbid older individual: A qualitative study. *Int J Clin Pract* 2016;70:261-76. doi:10.1111/ijcp.12780

31 Sippli K, Rieger MA, Huettig F. GPs' and dentists' experiences and expectations of interprofessional collaboration: Findings from a qualitative study in Germany. *BMC Health Serv Res* 2017;17:179. doi:10.1186/s12913-017-2116-4

32 Merle B, Haesebaert J, Bedouet A, et al. Osteoporosis prevention: Where are the barriers to improvement in French general practitioners? A qualitative study. *PLoS One* 2019;14:e0219681.

33 Merle B, Dupraz C, Haesebaert J, et al. Osteoporosis prevention: where are the barriers to improvement in a French general population? A qualitative study. *Osteoporos Int* 2019;30:177–85. doi:10.1007/s00198-018-4720-5

34 Drew S, Judge A, Cooper C, et al. Secondary prevention of fractures after hip fracture: a qualitative study of effective service delivery. *Osteoporos Int* 2016;27:1719-27. doi:10.1007/s00198-015-3452-z

35 Alami S, Hervouet L, Poiraudeau S, et al. Barriers to effective postmenopausal osteoporosis treatment: A qualitative study of patients' and practitioners' views. *PLoS One* 2016;11. doi:10.1371/journal.pone.0158365

36 Guzman-Clark JRS, Fang MA, Sehl ME, et al. Barriers in the management of glucocorticoid-induced osteoporosis. *Arthritis Care Res* 2007;140-6. doi:10.1002/art.22462

37 Lau E, Papaioannou A, Dolovich L, et al. Patients' adherence to osteoporosis therapy: Exploring the perceptions of postmenopausal women. *Can Fam*

For peer review only

Physician 2008;54:394-402.

38 Salter C, McDaid L, Bhattacharya D, et al. Abandoned Acid? Understanding adherence to bisphosphonate medications for the prevention of osteoporosis among older women: A qualitative longitudinal study. *PLoS One* 2014;9. doi:10.1371/journal.pone.0083552

39 Iversen MD, Vora RR, Servi A, et al. Factors affecting adherence to osteoporosis medications: A focus group approach examining viewpoints of patients and providers. *J. Geriatr. Phys. Ther* 2011;34:72. doi:10.1097/JPT.0b013e3181ff03b4

40 Mazor KM, Velten S, Andrade SE, et al. Older womens views about prescription osteoporosis medication: A cross-sectional, qualitative study. *Drugs and Aging* 2010;27:999-1008. doi:10.2165/11584790-000000000-00000

41 Wozniak LA, Johnson JA, McAlister FA, et al. Understanding fragility fracture patients' decision-making process regarding bisphosphonate treatment. *Osteoporos Int* 2017;28:219-29. doi:10.1007/s00198-016-3693-5

42 Swart KMA, Van Vilsteren M, Van Hout W, et al. Factors related to intentional non-initiation of bisphosphonate treatment in patients with a high fracture risk in primary care: A qualitative study. *BMC Fam Pract* 2018;19:141. doi:10.1186/s12875-018-0828-0

43 Scoville EA, de Leon Lovaton PP, Shah ND, et al. Why do women reject bisphosphonates for osteoporosis? a videographic study. *PLoS One* 2011;6. doi:10.1371/journal.pone.0018468

44 Sale JEM, Gignac MA, Hawker G, et al. Decision to take osteoporosis medication in patients who have had a fracture and are 'high' risk for future fracture: A qualitative study. *BMC Musculoskelet Disord* 2011;12:92. doi:10.1186/1471-2474-12-92

45 Sturrock A, Preshaw P, Hayes C, et al. Attitudes and perceptions of GPs and community pharmacists towards their role in the prevention of bisphosphonate-related osteonecrosis of the jaw: A qualitative study in the North East of England. *BMJ Open* 2017;7:e016047. doi:10.1136/bmjopen-2017-016047

For peer review only

46 Sturrock A, Preshaw PM, Hayes C, et al. Perceptions and attitudes of patients towards medication-related osteonecrosis of the jaw (MRONJ): A qualitative study in England. *BMJ Open* 2019;9:e024376. doi:10.1136/bmjopen-2018-024376

47 Nunes V, Neilson J, O'flynn N, et al. Medicines adherence: involving patients in decisions about prescribed medicines and supporting adherence (CG76). *NICE Guidance* 2009.

48 Horne R, Cooper V, Wileman V, et al. Supporting Adherence to Medicines for Long-Term Conditions. *Eur Psychol* 2019. doi: 10.1027/1016-9040/a000353

49 Driesenaar JA, De Smet PA, van Hulten R, et al. Beliefs about inhaled corticosteroids: comparison of community pharmacists, pharmacy technicians and patients with asthma. *J. Asthma* 2016;53:1051-8.

50 Checkland K, Harrison S, Marshall M. Is the metaphor of 'barriers to change' useful in understanding implementation? Evidence from general medical practice. *J Heal Serv Res Policy* 2007;12:95-00. doi:10.1258/135581907780279657

51 Beauvais, C., Poivret, D., Lespessailles, E. et al. Understanding Patients' Perspectives and Educational Needs by Type of Osteoporosis in Men and Women and People with Glucocorticosteroid-Induced Osteoporosis: A Qualitative Study to Improve Disease Management. *Calcif Tissue Int* 105, 589–608 (2019). https://doi.org/10.1007/s00223-019-00607-z

For peer review only

Figure legends

Figure 1. Prisma Diagram

Figure 2. Identified themes and subthemes mapped to the Theoretical Framework of Acceptability (TFA)

For peer review only

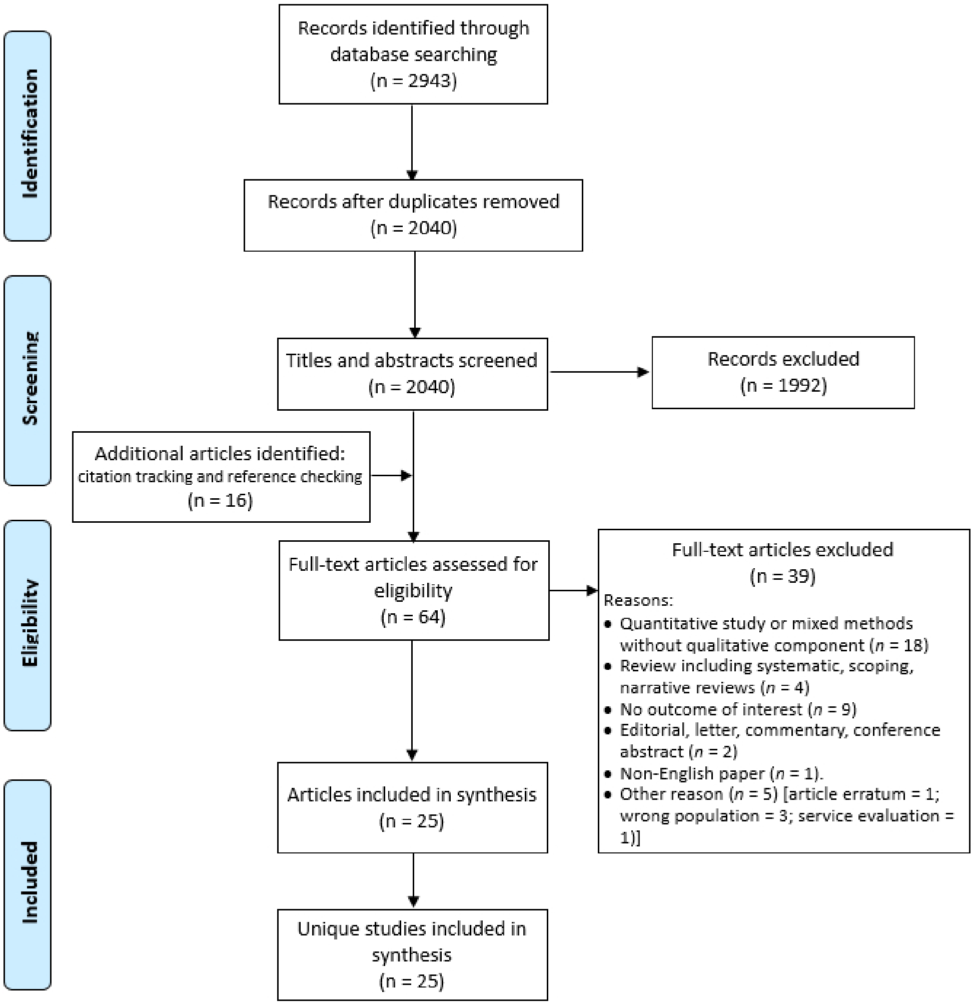

Prisma Diagram

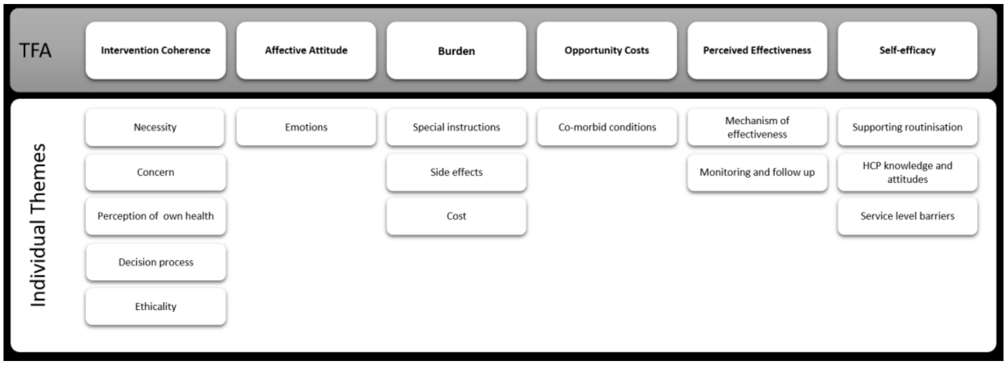

Identified themes and subthemes mapped to the Theoretical Framework of Acceptability (TFA)

Supplementary Table 1. PRISMA 2009 Checklist

Section/topic	#	Checklist item	Reported on page #
**TITLE**			
Title	1	Identify the report as a systematic review, meta-analysis, or both.	1
**ABSTRACT**			
Structured summary	2	Provide a structured summary including, as applicable: background; objectives; data sources; study eligibility criteria, participants, and interventions; study appraisal and synthesis methods; results; limitations; conclusions and implications of key findings; systematic review registration number.	3
**INTRODUCTION**			
Rationale	3	Describe the rationale for the review in the context of what is already known.	4-5
Objectives	4	Provide an explicit statement of questions being addressed with reference to participants, interventions, comparisons, outcomes, and study design (PICOS).	5
**METHODS**			
Protocol and registration	5	Indicate if a review protocol exists, if and where it can be accessed (e.g., Web address), and, if available, provide registration information including registration number.	5
Eligibility criteria	6	Specify study characteristics (e.g., PICOS, length of follow-up) and report characteristics (e.g., years considered, language, publication status) used as criteria for eligibility, giving rationale.	5-6
Information sources	7	Describe all information sources (e.g., databases with dates of coverage, contact with study authors to identify additional studies) in the search and date last searched.	6
Search	8	Present full electronic search strategy for at least one database, including any limits used, such that it could be repeated.	Supplementary material
Study selection	9	State the process for selecting studies (i.e., screening, eligibility, included in systematic review, and, if applicable, included in the meta-analysis).	6-7
Data collection process	10	Describe method of data extraction from reports (e.g., piloted forms, independently, in duplicate) and any processes for obtaining and confirming data from investigators.	7
Data items	11	List and define all variables for which data were sought (e.g., PICOS, funding sources) and any assumptions and simplifications made.	7

For peer review only

Risk of bias in individual studies	12	Describe methods used for assessing risk of bias of individual studies (including specification of whether this was done at the study or outcome level), and how this information is to be used in any data synthesis.	7
Summary measures	13	State the principal summary measures (e.g., risk ratio, difference in means).	7
Synthesis of results	14	Describe the methods of handling data and combining results of studies, if done, including measures of consistency (e.g., I^2) for each meta-analysis.	7-9
Risk of bias across studies	15	Specify any assessment of risk of bias that may affect the cumulative evidence (e.g., publication bias, selective reporting within studies).	9
Additional analyses	16	Describe methods of additional analyses (e.g., sensitivity or subgroup analyses, meta-regression), if done, indicating which were pre-specified.	9
**RESULTS**			
Study selection	17	Give numbers of studies screened, assessed for eligibility, and included in the review, with reasons for exclusions at each stage, ideally with a flow diagram.	9
Study characteristics	18	For each study, present characteristics for which data were extracted (e.g., study size, PICOS, follow-up period) and provide the citations.	11-12
Risk of bias within studies	19	Present data on risk of bias of each study and, if available, any outcome level assessment (see item 12).	12-14
Results of individual studies	20	For all outcomes considered (benefits or harms), present, for each study: (a) simple summary data for each intervention group (b) effect estimates and confidence intervals, ideally with a forest plot.	12-14
Synthesis of results	21	Present results of each meta-analysis done, including confidence intervals and measures of consistency.	16-19
Risk of bias across studies	22	Present results of any assessment of risk of bias across studies (see Item 15).	16
Additional analysis	23	Give results of additional analyses, if done (e.g., sensitivity or subgroup analyses, meta-regression [see Item 16]).	16-17
**DISCUSSION**			
Summary of evidence	24	Summarize the main findings including the strength of evidence for each main outcome; consider their relevance to key groups (e.g., healthcare providers, users, and policy makers).	20-21
Limitations	25	Discuss limitations at study and outcome level (e.g., risk of bias), and at review-level (e.g., incomplete retrieval of identified research, reporting bias).	22
Conclusions	26	Provide a general interpretation of the results in the context of other evidence, and implications for future research.	22-23
**FUNDING**			

For peer review only

Funding	27	Describe sources of funding for the systematic review and other support (e.g., supply of data); role of funders for the systematic review.	23

For peer review only

Supplementary Material 1. OVID MEDLINE Search strategy

For Ovid: The following table is an explanation of the symbols used in the search strategy below.

/	indicates an index term (MeSH/EMTREE heading).
exp	before an index term indicates that all subheadings were selected.
af.	Indicates a search for a term in all fields.
.ti,ab,kf.	indicates a search for a term in title/abstract/word(s) in keyword [MEDLINE].
mp.	indicates a search for a term in 'multi-purpose' fields, including the title, abstract, floating sub-heading word, keyword heading word, subject heading word.
tw.	Indicates a search for a term in title and abstract.
$	at the end of a term indicates that this term has been truncated.
?	optional wild card character replaces zero or one character within a word or at the end of a word
adj	indicates a search for tw.o terms where they appear adjacent to each another
adj*n*	indicates a search for two terms where they appear within *n* words of each another

	Searches
1	diphosphonates/ or alendronate/ or ibandronic acid/ or risedronic acid/ or zoledronic acid/ or etidronic acid/ or pamidronate/
2	diphosphon$.ti,ab,kf.
3	bisphosphon$.ti,ab,kf.
4	alendron$.ti,ab,kf.
5	fosamax.ti,ab,kf.
6	risedron$.ti,ab,kf.
7	actonel.ti,ab,kf.
8	zoledron$.ti,ab,kf.
9	aclasta.ti,ab,kf.
10	ibandron$.ti,ab,kf.
11	etidron$.ti,ab,kf.
12	pamidron$.ti,ab,kf.
13	or/1-12
14	attitude/
15	attitude of health personnel/
16	exp attitude to health/ [includes patient satisfaction and patient preference]
17	choice behavior/
18	decision making/
19	attitud$.ti,ab,kf.
20	percept$.ti,ab,kf.
21	expectation$.ti,ab,kf.

22	experienc$.ti,ab,kf.
23	preferen$.ti,ab,kf.
24	choice$.ti,ab,kf.
25	belie$.ti,ab,kf.
26	opinion$.ti,ab,kf.
27	priorit$.ti,ab,kf.
28	benefi$.ti,ab,kf.
29	reason$.ti,ab,kf.
30	decision$.ti,ab,kf.
31	motiv$.ti,ab,kf.
32	justif$.ti,ab,kf.
33	(concern or concerns or concerned).ti,ab,kf.
34	(view or views or viewed).ti,ab,kf.
35	satisf$.ti,ab,kf.
36	value$1.ti,ab,kf.
37	or/14-36
38	Qualitative Research/ [After DeJean et al., 2016. Qual Health Res **26**(10): 1307-1317]
39	interview/
40	(theme$ or thematic).mp.
41	qualitative.af.
42	nursing methodology research/
43	questionnaire$.mp.
44	ethnological research.mp.
45	ethnograph$.mp.
46	ethnonursing.af.
47	phenomenol$.af.
48	(grounded adj (theor$ or study or studies or research or analys?s)).af.
49	(life stor$ or women$ stor$).mp.
50	(emic or etic or hermeneutic$ or heuristic$ or semiotic$).af.
51	((data adj1 saturat$) or participant observ$).tw.
52	(social construct$ or postmodern$ or post modern$ or poststructural$ or post structural$ or feminis$ or interpret$).mp.
53	(action research or cooperative inquir$ or co operative inquir$).mp.
54	(humanistic or existential or experiential or paradigm$).mp.

55	(field adj (study or studies or research)).tw.
56	human science.tw.
57	biographical method.tw.
58	theoretical sampl$.af.
59	((purpos$ adj4 sampl$) or (focus adj group$)).af.
60	(account or accounts or unstructured or open ended or text$ or narrative$).mp.
61	(life world or conversation analys?s or personal experience$ or theoretical saturation).mp.
62	((lived or life) adj experience$).mp.
63	cluster sampl$.mp.
64	observational method$.af.
65	content analysis.af.
66	(constant adj (comparative or comparison)).af.
67	((discourse$ or discurs$) adj3 analys?s).tw.
68	narrative analys?s.af.
69	heidegger$.tw.
70	colaizzi$.tw.
71	spiegelberg$.tw.
72	van manen$.tw.
73	van kaam$.tw.
74	merleau ponty.tw.
75	husserl$.tw.
76	foucault$.tw.
77	(corbin$ adj2 strauss$).tw.
78	glaser$.tw.
79	(mix$ adj2 (method$ or design$)).af. [filter amended to identify mixed method studies]
80	or/38-79
81	13 and 37 and 80

Supplementary Material 2. CASP Quality Appraisal Checklist

All ten questions answered with one of four options: Yes, unsure, partial, or No

Section A: Are the results of the study valid?

1. Was there a clear statement of the aims of the research?
2. Is a qualitative methodology appropriate?
3. Was the research design appropriate to address the aims of the research?
4. Was the recruitment strategy appropriate to the aims of the research?
5. Was the data collected in a way that addressed the research issue?
6. Has the relationship between researcher and participants been adequately considered?

Section B: What are the results?

7. Have ethical issues been taken into consideration?
8. Was the data analysis sufficiently rigorous?
9. Is there a clear statement of findings?

Section C: Will the results help locally?

10. How valuable is the research?

For peer review only

Supplementary Material 3. Subtheme descriptions and illustrative key findings

Main theme	Subtheme	Description	Illustrative Key findings
Intervention Coherence	Necessity	Both patient and clinician participants described osteoporosis, falling and fracturing as a normal part of ageing and this view was associated with the perception that medication or treatment was futile.[16,38] One GP described the 'problem is not with the treatment, it's with the diagnosis': perceiving that the indications for treatment had broadened over recent years.[42] The absence of symptoms was reported by clinicians as a disincentive to patients accepting treatment,[33,36] however, patients questioned whether osteoporosis really was asymptomatic.[23] Patient participants who conceptualised osteoporosis as having consequences, e.g. as a cause of disability including 'shrinking' and 'stooping', were motivated to take medication.[37] Patient participants described other ways of controlling their condition and preventing fracture, for example, by not falling.[35]<br>In some patients who initiated treatment, the notion of osteoporosis as a chronic disease was noted not to make sense with the need to take bisphosphonate medication for 5 years.[23]	Patients perceived minimal susceptibility to the negative consequence of osteoporosis in the future and did not consider osteoporosis to be a serious health condition.[30]<br>Avoiding consequences (including shrinking, stooping, fractures) of osteoporosis was a strong motivator for adherence in PMW.[37]
	Concerns	Before starting bisphosphonates, patients noted concern and fear of bisphosphonate-specific side effects. This could be informed by vicarious experience of a family member,[43] or information from the media.[37] The special instructions for use, the limited duration of treatment and the name 'acid' were all cited as reasons underlying the perception that bisphosphonates must be harmful. Both patients and HCP's also cited a mistrust of pharmaceutical companies,[33,38,42] or a general aversion to drugs.[35,37,39,42]	{Women} were concerned about the long lists of drug side effects in advertisements.[16]<br>'Once you're on it, then it stays in your system and you wonder what damage have you've done to yourself?'[16]<br>Some PMW did not like the idea of taking any medications because they viewed medications as artificial and thought they had unpredictable effects.[37]
	Perceptions of own health	Some patients reported a perception that they were healthy, with some disbelieving they had osteoporosis and/or high fracture risk, and therefore and would reject medication and a label of a disease.[37] Conversely, others conceptualised bisphosphonates as a	Some patients initiated bisphosphonates to stay healthy.[41]<br>For PMW who considered themselves healthy, the idea of

For peer review only

		mechanism to remain healthy[41] and/or autonomous.[38] In a study of French GPs, on respondent also suggested patients wanted to know how to "'age well".[33]	medication was disconcerting as it meant perceiving themselves as sick.[37]
	Decision process	Across studies patients and HCPs described perceptions that the benefits did not outweigh the risks.[16,29,35,41,] Often in these descriptions, the value of treatment was not clearly articulated meaning this assessment meant the patient weighing up staying as they were, or experiencing new side effects.[38] However, even when the risk of fracture was acknowledged, medication could still be seen as something to avoid.[35] The opposing view that the 'benefits were worth the costs' was evident in circumstances where benefits were described.[37] Others studies with patients reported that this decision was 'difficult' with one participant describing it as like 'Russian roulette'.[44]<br>Balancing necessity against concerns was influenced by contingent factors such as trust in the clinician and could either be an easy or difficult and ongoing process. Patient participants talked about 'confidence' and 'trust' in their HCP, which could be associated with minimal contemplation to take treatment, or alternatively mistrust, or a failure to be 'convinced'.[16,25,26,37,40] Some patients reported clinicians as being persistent in their recommendation to take bisphosphonates;[40] however, conversely, patients also described by dissuaded by their doctor against treatment.[32] Often, patients described seeking information from other sources to make the final decision which often resulted in a decision against treatment.[44]<br>For those who initiated medication, an ongoing re-assessment of risk and benefit was noted,[23,41,44] particularly in studies that employed longitudinal methods.[27,38, 41] Patients reported their decision making was influenced by experiencing a future fracture,[44] follow-up scans,[25] experienced side effects,[37,38] views of others and other experienced illnesses or life events.[27]	For some, the decision to take bisphosphonate involved minimal contemplation because they liked/trusted their health care provider.[44]<br>Patients who found the decision difficult sought alternative sources of information (professional and non) which often resulted in decision not to take OP medication.[44]
	Ethicality	Both orthopaedic and primary care clinicians reported a 'bias' against treating the elderly due to a belief 'nothing can be done for them'.[16] However, some patients also perceived that they were too old to benefit.[35] HCPs were seen to use the using ethical principle of non-maleficence to justify not recommending bisphosphonates. They questioned the negative side effects 'for a benefit that has not really been proven' and worried about being blamed for causing their patients ill-health.[28,40] Patients, in some circumstances, doubted the beneficence of the health care professionals e.g. perceiving	Clinicians {primary care and specialists} report bias against treating elderly patients because of a general tendency to believe that nothing can be done for them.[16]

For peer review only

		their physician as a 'pill pusher' or the motivation for prescribing medication being to receive money in return.[40]	
Affective Attitudes	Emotions	Patients described wide-ranging fears including fear of common and rare side effects and fear of new side effects emerging in the future. Patients described fear of bisphosphonates staying in their system,[16] with one patient participant describing bisphosphonates as akin to chemicals used to clean machines.[23] Patients also worried information was being withheld, or were fearful of the sheer amount of information to take in.[37] Both clinicians and patients described media reports as the source of fear, with patients also citing experiences of friends and family.[37] Fear of addiction was mentioned by patients in one study.[23] Patients and HCPs also expressed annoyance with the special instructions associated with oral bisphosphonate use, and annoyance with experienced oesophageal side effects.[40]<br>In two studies, patient participants reported that they experienced feelings of safety and reassurance when taking bisphosphonates,[26] linked to the anticipated benefits.[37]	"..when I read the side effects it was like a *horror film* really".[38]<br><br>medication provided a feeling of safety and reassurance.[26]
Burden	Special instructions	The method of administration of oral bisphosphonates caused concern to patients, both prior to initiating treatment,[42] and whilst on the treatment,[32] causing disruption to daily life. The need to remain upright after taking the medication and only being allowed to drink water was burdensome, and led to some disregarding the administration requirements.[37] Specific activities that needed to be actioned first thing in the morning also competed with taking oral bisphosphonates, with patients citing examples such as the need to have a coffee or run a family errand early every morning.[44] Primary care physicians reported that taking bisphosphonates was a 'hassle' for patients.[16] The frequency of the oral bisphosphonates, once a week, led to a number of reports of patients forgetting to take their medication.[16,23,37-39] Varying reports were identified about whether daily or weekly regimes were more or less burdensome.[16,37] Four studies reported patients' perceptions that the special instructions were not disruptive or burdensome.[26,27,37,39]	Some patients were able to rearrange their daily routines to accommodate {bisphosphonate} requirements, but others would intentionally disregard the administration requirements or forget to take the medication if it did not fit into their schedules.[37]
	Side effects	Experienced side effects were discussed in three of the studies interviewing clinicians,[28,29,31] eight with patients[23,26,27,37,38,41,44,45] and five with mixed participants.[16,35,39,42,43] Experienced side effects were reported as a common reason for lack of adherence, with gastrointestinal disturbances being described as	Gastrointestinal disturbances from taking bisphosphonates were most notable and were described as

For peer review only

		"horrendous diarrhoea" and "wrecking my stomach.[37,39] Patients reported stopping medications after experiencing side effects, did not always disclose side effects to HCPs and noted that the treatment 'was almost more disabling than the disease'.[27,32,46]	"horrendous diarrhoea" and "wrecking my stomach.[37]
	Costs	Financial costs were discussed in five studies, four of which were conducted in North America and one in Australia.[16,28,37,43,46] Patients did not report cost as a barrier to bisphosphonates specifically, however, medical insurance was perceived by clinicians as a barrier due to its complexity.[29,39,43] Indirect costs relating to travel and the need for increased dental checks were mentioned briefly but not described as a problem.[45,46]	Cost was not a limiting factor to adherence if patients had insurance coverage for medications. Even patients without insurance expressed a willingness to make sacrifices to pay for the medications because they thought the benefits were worth the cost.[37] Providers {secondary care} stated that due to cost not being covered by insurance companies, patients stop taking or alter dose/frequency.[39]
Opportunity costs	Co-morbid conditions	Physicians perceived bisphosphonate treatment was less important to patients who might have other more pressing health conditions [29,45] particularly in the absence of symptoms.[27,33] Patients also reported that other health conditions took priority over their prescribed bisphosphate leading them not to start or discontinue medication.[32] Within the time-limited consultation, multiple competing priorities relating to other health conditions was reported by HCPs, resulting in a 'pecking order', and less time to discuss bisphosphonates.[35,45]	(Bisphosphonates) are lower down in the pecking order of things that we look at when we are supervising polypharmacy, when we are looking at chronic disease management".[45]
Perceived effectiveness	Mechanism of effectiveness	*Mechanism of effectiveness:* Patients expressed confusion about how bisphosphonates work and uncertainty about whether they strengthen, prevent worsening or slow the decline in bone density.[25,26,39] Patients talked about bone density scans as providing 'proof' of whether their medication was effective, however, there were differing reports of whether stabilisation in density was considered as treatment success.[35,40] The lack of systematic reduction in fracture or improvement in bone density was noted to result in ambivalence about efficacy and importance.[35] Patients described wanting more explanation about, and evidence of effectiveness (including quantified benefit).[16,23,37,38,40] Prior to initiating treatment, the perceived effectiveness of bisphosphonates was influenced in patients primarily by vicarious experience of friends	Taking anti-osteoporosis drugs was noted to not always seem to lead to improvement in their bone density and did not systematically prevent fracture.[35]

For peer review only

		or relatives.[40,42,43] Examples of relatives who had fractured on treatment or had hip or knee joint replacements were given as examples of lack of efficacy.[42] Patients cited clinicians not meeting their informational needs about effectiveness, which may have been due to their own reported doubts.[29,42] Other clinicians expressed continued doubts about effectiveness in specific populations (e.g. the elderly) or in in relation to fracture risk at specific sites.[35] Patients in one study reported being told by health care professionals bisphosphonates are not effective for everyone[24] and in one study, clinicians questioned predictors of response.[29]	
	Monitoring and follow-up	Follow-up and monitoring were reported by clinicians[34] and patients[38] to support adherence to oral treatment, but generally felt to be lacking in primary care, in part due to uncertainties about who, when and what to monitor.[34] Patients reported not feeling supported with continued persistence with treatment[38] and reported the need for more reviews, feedback and help with 'ways to keep going' with medications.[16,23,38]	Women anticipated the next DXA scan as being the "proof" of whether the treatment was effective.[2731] Reviewing patients' BMD results with them helped them evaluate the status of their osteoporosis, which motivated them to either start or continue taking their medicine.[37]
Self-efficacy	Supporting routinisation	*Supporting routinisation* Being able to successfully follow the special instructions for taking oral bisphosphonates, and incorporate the regime into daily routines appeared to be important to acceptability.[39] Other reported strategies to support self-efficacy were using pill compartments and calendar systems/reminders.[16] Patients reported that HCPs should supplement their oral instructions about BP administration with written ones.[39] Information, support and encouragement was needed throughout treatment but felt to be lacking by patients[16,38,44]. Patients and HCPs reported insufficient time in consultations to cover all the information about bisphosphonate medication.[35,39]	Patients noted that tips for routinizing medication use, such as using triggers (e.g., meals, calendars, placement of medications) to remember when to take medications, facilitated long-term adherence.[16]
	HCP knowledge and attitudes	Primary care providers did not feel confident in their own knowledge about bisphosphonates; they described guidelines as confusing and too detailed, expressing a number of uncertainties relating to who to start medication in, how long to continue medication for, the relationship between bisphosphonates and co-dependency for calcium/vitamin D, safety, when treatment should be changed including dose.[16,25,29,35] Some primary care clinicians indirectly suggested perceptions that osteoporosis was not a priority. Secondary care providers suggested osteoporosis champions in primary care would help educate primary care clinicians who were less interested in the condition.[39,45] It was also reported that non-medical clinicians	Physicians reported need for training in treating and help with therapeutic decision making.[35]

For peer review only

		(pharmacists or nurses) may be more knowledgeable or have more time to discuss bisphosphonates.[39,45]	
	Service level barriers	In terms of professional roles, clinicians in two studies described uncertainty about whose role it was to start and monitor treatment.[16,34] This was compounded by perceived poor communication between primary and secondary care, including update of the patients prescriptions on the electronic medical record.[39] Further reported barriers to treatment included lack of incentivisation[34] difficulty ordering, accessing or interpreting investigations to monitor treatment,[16,29] external restrictions on prescribing and access to intravenous bisphosphonates[34] and lack of time in primary care consultations.[16]	Provider barriers to treatment include lack of knowledge, other priorities, limited access and limited time.[36]<br>GPs regretted the absence of consensus about the professional in charge of osteoporosis.[32]<br>A number of participants {HCPs/managers} thought that intravenous zoledronic acid should be more widely available to improve adherence.[34]

For peer review only
